# Monocytes of patients with familial hypercholesterolemia show alterations in cholesterol metabolism

**DOI:** 10.1186/1755-8794-1-60

**Published:** 2008-11-28

**Authors:** Sandy Mosig, Knut Rennert, Petra Büttner, Siegfried Krause, Dieter Lütjohann, Muhidien Soufi, Regine Heller, Harald Funke

**Affiliations:** 1 Molecular Hemostaseology, Friedrich-Schiller-University of Jena, Bachstrasse 18, 07743 Jena, Germany; 2 Institute of Molecular Cell Biology, Friedrich-Schiller-University of Jena, Leutragraben 3, 07743 Jena, Germany; 3 Institute of Clinical Biochemistry and Pharmacology, Rheinische Friedrich-Wilhelms-University Bonn, Sigmund-Freud-Str.25, 53105 Bonn, Germany; 4 Department of Cardiology, Philipps-University Marburg, Baldingerstrasse 1, 35043 Marburg, Germany

## Abstract

**Background:**

Elevated plasma cholesterol promotes the formation of atherosclerotic lesions in which monocyte-derived lipid-laden macrophages are frequently found. To analyze, if circulating monocytes already show increased lipid content and differences in lipoprotein metabolism, we compared monocytes from patients with Familial Hypercholesterolemia (FH) with those from healthy individuals.

**Methods:**

Cholesterol and oxidized cholesterol metabolite serum levels of FH and of healthy, gender/age matched control subjects were measured by combined gas chromatography – mass spectroscopy. Monocytes from patients with FH and from healthy subjects were isolated by antibody-assisted density centrifugation. Gene expression profiles of isolated monocytes were measured using Affymetrix HG-U 133 Plus 2.0 microarrays. We compared monocyte gene expression profiles from FH patients with healthy controls using a Welch T-test with correction for multiple testing (p < 0.05; Benjamini Hochberg correction, False Discovery Rate = 0.05). The differential expression of FH associated genes was validated at the mRNA level by qRT-PCR and/or at the protein level by Western Blot or flow cytometry. Functional validation of monocyte scavenger receptor activities were done by binding assays and dose/time dependent uptake analysis using native and oxidized LDL.

**Results:**

Using microarray analysis we found in FH patients a significant up-regulation of 1,617 genes and a down-regulation of 701 genes compared to monocytes from healthy individuals. These include genes of proteins that are involved in the uptake, biosynthesis, disposition, and cellular efflux of cholesterol. In addition, plasma from FH patients contains elevated amounts of sterols and oxysterols. An increased uptake of oxidized as well as of native LDL by FH monocytes combined with a down-regulation of NPC1 and ABCA1 explains the lipid accumulation observed in these cells.

**Conclusion:**

Our data demonstrate that circulating FH monocytes show differences in cell physiology that may contribute to the early onset of atherosclerosis in this disease.

## Background

Atherosclerosis is the primary cause of coronary heart disease (CHD) and stroke in Western societies [1]. It is characterized by the development of lipid-rich lesions in the arterial wall, in which foam cells, monocyte-derived lipid-laden macrophages, are frequently found.

An important regulator of cellular cholesterol content is the sterol regulatory element binding protein (SREBP) pathway, which controls, by transcriptional regulation, the uptake of cholesterol via LDL-receptor and several steps in the *de novo *synthesis of cholesterol.

In healthy individuals cells ingest cholesterol by endocytosis of LDL bound to the LDL-receptor (LDLR). After endocytosis, the LDLR uncouples from its ligand and returns to the cell surface, while the LDL is catabolized. Cholesterol accumulation in membranes of the endoplasmatic reticulum (ER) results in a down-regulation of the SREBP pathway and subsequently in the repression of 3-hydroxy-3-methyl-glutaryl-CoA-reductase (Hmgcr), the rate limiting enzyme of the *de novo *cholesterol biosynthesis [2]. Efflux of excessive cholesterol is mediated by Abca1, the major cholesterol efflux system in macrophages, which transfers cholesterol to apolipoprotein A1 on HDL particles. It is assumed that Npc1 has a pivotal role in cholesterol efflux as it aids in the cellular distribution of lysosomal cholesterol which enables Abca1 dependent efflux [3,4].

In contrast to native LDL, macrophages utilize scavenger receptors (SR), such as CD36, for the uptake of modified LDL [5]. Several studies have demonstrated that macrophages express high levels of CD36 enabling them to bind and internalize oxidized lipoproteins. It is well established that oxidized LDL (oxLDL) is cytotoxic and that it has the ability to induce apoptosis. As oxLDL is frequently found in atherosclerotic lesions it is assumed that its accumulation contributes to the pathogenesis of atherosclerosis [5]. Therefore, a rapid clearance of oxLDL deposits from the arterial wall via SRs by monocyte derived macrophages is essential for atherosclerosis prevention.

However, only few data exist on the involvement of circulating monocytes in this pathologic process in men [6]. To date, studies of atherosclerosis development have been carried out mostly in pathologic human specimen, cell lines, and primary cell culture systems, as well as in animal models [5]. To study monocyte function in a hyperlipidemic environment in men we examined patients suffering from the monogenic disorder Familial Hypercholesterolemia (FH). FH patients have a defective or missing LDLR which results in dramatically elevated plasma LDL-cholesterol levels and early onset of atherosclerosis. Further, atherosclerosis progression in FH is mainly independent from the presence of additional genetic and environmental risk factors [7] which makes it a suitable model trait for studying human atherosclerosis.

## Methods

### Participants

Homozygous and heterozygous FH patients with documented genetic defects in the LDL receptor (LDLR) gene and healthy volunteers were informed about the aim of the study and gave written informed consent. The study was approved by the Ethics Committee of the Friedrich Schiller University of Jena/Germany. All homozygous FH patients were treated with haemapheresis. In addition, three of eight homozygous patients also received statin treatment. Of the heterozygous patients three received neither statin nor apheresis therapy. Five heterozygous patients received apheresis as well as statin therapy. Those patients who did not receive statin treatment did not show a significant response in their plasma cholesterol levels to the drug treatment. (FH patients: LDLR mutations given in Table 4).

### Cell isolation, cryopreservation and cell culture

For microarray analysis and cell culture assays cells were isolated with RosetteSep Monocyte Enrichment Cocktail (Cell Systems, St. Katharinen, Germany) according to the manufacturer's protocol. Cell purity was analyzed by flow cytometry with antibodies directed against CD14 (monocytes), CD3 (T cells), CD235a (erythrocytes), CD19 (B cells), and CD56 (NK cells). All cell preparations had a purity of > 95%. The viability of cells was checked by staining with 7-amino-actinomycin D (7-AAD) (Becton Dickinson, Heidelberg, Germany). The number of 7-AAD positive cells was below 3% in all preparations. In addition, the viability of monocytes was confirmed by their inflammatory response to LPS (Sigma-Aldrich, Munich, Germany) stimulation (data not shown).

For cell culture assays, isolated monocytes were resuspended in 10% DMSO (Sigma-Aldrich, Munich, Germany), 60% IMDM (Invitrogen, Karlsruhe, Germany), and 30% autologous serum. They were then frozen with a temperature decline of -1°C/min to -80°C, and stored in liquid nitrogen. Frozen cells were thawed at 37°C, washed twice with PBS/2 mM EDTA and resuspended in X-VIVO 15 (Cambrex, Walkersville, USA). For all binding, uptake and blocking assays 1 × 10^5 ^monocytes per well were cultured in X-VIVO 15 serum free medium with the indicated concentrations of nLDL, oxLDL, and antibodies.

Monocyte to macrophage differentiation was carried out by culturing cryopreserved monocytes for 10 days in X-VIVO 15 medium (Lonza, Walkersville, MD) with addition of 10% of human serum from a healthy, normolipidemic donor.

### Microarray analysis

Freshly isolated cells were lysed in TRIZOL (Invitrogen, Karlsruhe, Germany). RNA was extracted using the RNeasy Micro Kit (Qiagen, Hilden, Germany). RNA was quantified using a Nanodrop photometer (Thermo Scientific, Wilmington, Delaware, USA). RNA quality was measured with an Agilent 2100 Bioanalyzer using the RNA Nano kit (Agilent Technologies, Waldbronn, Germany). Only RNA with a RNA integrity number better than 9.5 was used for microarrays. 1 μg of total RNA was processed with Affymetrix One-Cycle Target Labeling Control reagents and hybridized to Affymetrix HG U133 Plus 2.0 GeneChip Arrays. Raw data were obtained with Affymetrix GCOS 1.3. Quality control of microarray raw data was done with the Bioconductor "affyQCReport" R package including Whisker box plots, density plots, plotting of 3'/5' ratios of GAPDH and beta-actin, scaling factor and background noise, and correlation plots of all microarrays (Supplementary Figure 1). Raw data were imported to GeneSpring GX 7.3.1 (Agilent Technologies) using the RMA (Robust Multi-Array Average) algorithm [8]. They were normalized per chip to the 50^th ^percentile and per gene to the median. Transcripts with a raw data value of 20 and more in at least half of all measured arrays were considered as "expressed". 35.264 transcripts fulfilled this criterion. Functional annotation clustering was performed with DAVID 2008 . The resulting group enrichment score represents the negative logarithm of the geometric mean of Fisher's exact test p-values from cluster members [9,10]. Pathway analysis was done with the Agilent Pathway Architect (Agilent, Waldbronn, Germany).

Microarray data samples were deposited in the Gene Expression Omnibus database . They are accessible under the series GSE6054.

### Quantitative Real-time PCR

Quantitative Real-time PCR (qRT-PCR) was performed using the Eppendorf RealPlex 2S PCR (Eppendorf, Hamburg, Germany) and QuantiTect Primer Assays (Qiagen, Hilden, Germany). Quantification was done using standards with known copy numbers and normalization to ubiquitin C (UBC).

### LDL-isolation, preparation and labelling

LDL were isolated from fresh serum of normolipidemic donors by sequential ultracentrifugation [11] and stored in 0.02% NaN_3_, Na_2_EDTA (0.24 mM) at 4°C overlaid with nitrogen. Preparations were used within two weeks. Oxidation of LDL was achieved by a 6 hour incubation of native LDL with 10 μM CuSO_4 _at 37°C. Oxidized LDL was desalted by PD-10 Desalting columns from GE Healthcare (Uppsala, Sweden) and eluted in PBS.

nLDL and oxLDL were labelled using the Alexa-Fluor488- and Alexa-Fluor647-Protein labelling kits (Molecular Probes, Leiden, Netherlands) according to the manufacturer's protocols. All LDL forms were endotoxin-low (< 0.02 EU/ml), tested by QCL-1000 Chromogenic LAL (Cambrex, Walkersville, USA). The OxLDL ELISA-Kit was obtained from Immundiagnostik AG (Bensheim, Germany). LDL protein content was measured by a modified Lowry protein assay (Biorad, Hercules, USA).

### Oil red O staining

10^5 ^monocytes/well were seeded and adhered to coverslips (Nalge Nunc International, Rochester, USA) in X-VIVO 15 for 4 hours. After adhesion, cells were washed twice with PBS/2 mM EDTA and once with PBS. They were then fixed using 4% paraformaldehyd for 10 minutes, rinsed with 60% isopropanol, incubated for 10 minutes with Oil Red O, rinsed again with isopropanol, and washed with aqua dest. After hematoxylin-incubation and -oxidation coverslips were rinsed again and were imbedded in aqueous mounting medium. Images have been taken on Zeiss AxioVert200M (Carl Zeiss, Jena, Germany).

### Plasma-concentrations of sterols

10 μg/ml butylhydroxytoluol (Sigma Aldrich, Munich, Germany) were added to fresh plasma of donors which was subsequently frozen and stored at -80°C until analysis. Serum concentrations of cholesterol were measured by gas chromatography-flame ionization detection using 5alpha-cholestane as internal standard and cholesterol precursors and plant sterols by gas chromatography-mass spectrometry with epicoprostanol as internal standard. Oxidized cholesterol metabolites (7α-, 24*S*- and 27-hydroxycholesterol) were analyzed using an isotope dilution method and quantified by selected ion monitoring gas chromatography-mass spectrometry [12].

### Time and dose dependency, binding and blocking experiments

For uptake studies monocytes were washed as described above and incubated with LDL for the indicated times at 37°C, 5% CO_2_. Binding studies were performed at 4°C. CD36 and Lrp1 blocking was done with 10 μg/ml murine IgG (MOPC-21) (Abcam, Cambridge, UK), rabbit IgG (Innovative Research, Southfield, USA), anti-human CD36 (FA6-152) (Abcam, Cambridge, UK) or anti-human Lrp1 (Biomac, Leipzig, Germany) for 30 minutes at 37°C, 5% CO_2 _and incubated with the indicated LDL forms for 4 hours at 37°C, 5% CO_2_. All antibodies used were endotoxin-low and azide-free.

### Flow cytometry

Purity control antibodies, isotype control antibodies and anti-CD36 were from ImmunoTools (Friesoythe, Germany). Anti-CD91 was from BD Biosciences (Heidelberg, Germany). Flow cytometry has been performed on a FACSCalibur (Becton Dickinson, Heidelberg, Germany) and data analyzed with FlowJo 7.2.2. (TreeStar, Ashland, OR, USA).

### Western Blots

1 × 10^6 ^monocytes were lysed in lysis buffer (50 mM TrisCl pH 7.5, 150 mM NaCl, 5 mM EDTA, 1% NP-40, protease inhibitors). Protein content was measured using BCA-Protein-Assay Kit (Pierce, Rockford, USA). 10 μg protein/individual were subjected to SDS-PAGE and Western Blotting. Primary antibodies against β-Actin (Cell Signaling, Danvers, USA), ABCA1 (Novus Biologicals, Littleton, USA), SCAP, NPC1 (Santa Cruz Biotechnology, Heidelberg, Germany), CD36 (FA6-152) (Abcam, Cambridge, UK) and Lrp1 (R2629, kindly provided by DK Strickland) were used. Secondary horseradish-peroxidase conjugated goat anti-mouse, rabbit anti-goat and goat anti-rabbit antibodies were purchased from KPL (Gaithersburg, USA). ECL was from Perkin Elmer (Boston, USA).

### Statistical analyses

Statistical analyses were performed with SPSS 14.0 (SPSS, Chicago, USA) using two-sided Students t-Test, if not otherwise indicated. Error bars indicate the standard deviation.

## Results

**Figure 1:**
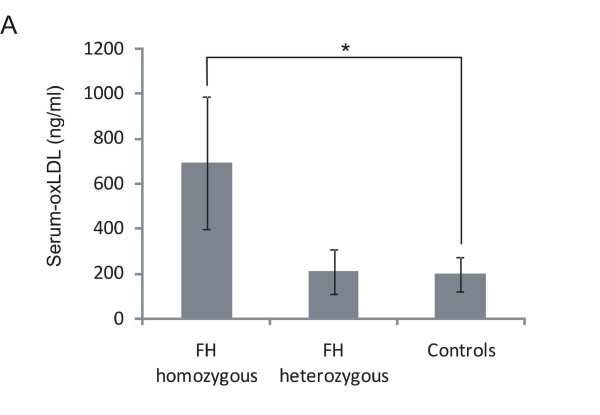
**Serum oxLDL levels and neutral lipid content of monocytes from FH patients and healthy individuals**. *A*: Serum oxLDL concentrations of homo- and heterozygous FH patients and controls (homozygous FH n = 8, heterozygous FH n = 8, controls n = 19). *B, C*: Monocytes from controls and homozygous FH patients were stained with Oil Red O to visualize neutral lipid content. *B*: Representative stainings are shown. *C*: Histogram of percentage of Oil Red O positive monocytes of FH patients and controls. Monocytes of 15 randomly taken pictures were counted, > 200 monocytes/person (FH homozygous n = 3, controls n = 4). Histogram bars indicate the mean ± SD, (* p < 0.05).

**Figure 2:**
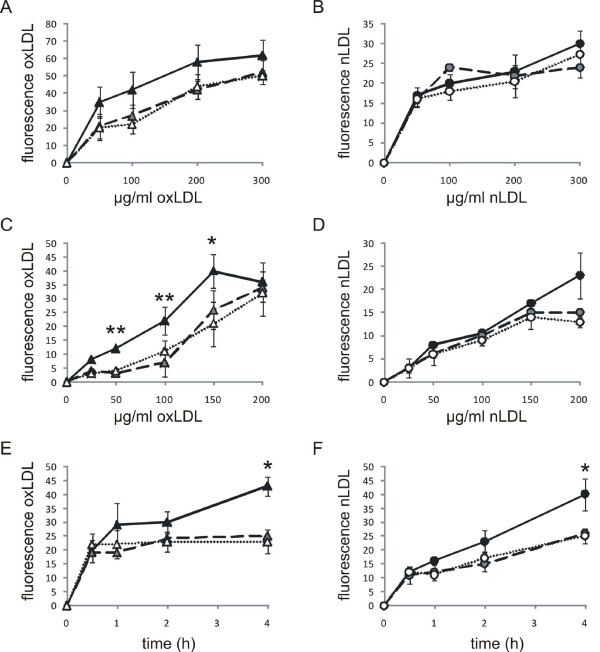
**LDL binding and uptake characteristics of monocytes of FH patients and healthy donors**. *A-E*: Binding and uptake of oxidized LDL (homozygous FH (black triangles) n = 4, heterozygous FH (gray triangles) n = 4 and healthy controls (open triangles) n = 4) and native LDL (homozygous FH (black circles) n = 4, heterozygous FH (gray circles), healthy controls (open circles) n = 4) by monocytes. *A*: Binding of AF488 labelled oxLDL and *B*: nLDL for 1 h at 4°C. *C*: Dose dependent uptake of AF488 labelled oxLDL and *D*: nLDL for 1 h at 37°C. *E*: Time dependent uptake of 100 μg/ml AF488 labelled oxLDL and *E*: nLDL for up to 4 h; please note that due to separate lipoprotein preparations for each experiment fluorescence intensities cannot be compared between subpanels A-E; comparison is only possible within a subpanel; experiments were done in duplicate; histogram bars indicate the mean ± SD (* p < 0.05, ** p < 0.01).

At first we measured plasma oxLDL levels of FH patients and healthy controls using ELISA, which were significantly increased in homozygous FH patients (Figure 1A, baseline characteristics given in Table 1). OxLDL levels in the plasma of heterozygous patients were not different from those of the control individuals. We also analyzed the plasma of FH patients and controls for sterols, including phyto- and oxysterols. All tested sterols, except the cholesterol biosynthesis intermediates lathosterol and lanosterol were significantly elevated in homozygous FH patients (Table 2). We then analyzed, if there were differences in the lipid content of monocytes from homozygous or heterozygous FH patients and controls using Oil Red O staining. Figures 1B, C [see Additional file 1] show that the number of Oil Red O positive cells is significantly higher in homozygous FH patients than in healthy donors, which may indicate an increased lipid content of FH monocytes. Heterozygous FH patients showed a slight increase in Oil Red O positive cells, however their number is not significantly different from those of control subjects. Although it would have been very interesting to directly measure intracellular cholesterol content limited amounts of sample material did not allow us to do the measurements. To identify potential sources of intracellular cholesterol we carried out LDL binding and LDL uptake studies and measured the binding of native LDL (nLDL) and oxidized LDL (oxLDL) (labelled with Alexa Fluor 488) after 1 hour incubation at 4°C using cryopreserved monocytes from FH patients and control individuals. Binding specificity was confirmed by a chase with a 50-fold excess of unlabelled LDL (data not shown). The binding capacity for oxLDL to homozygous FH monocytes was higher than in heterozygous FH monocytes and controls. The binding capacity for the latter ones was very similar (Figure 2A). Surprisingly, no binding difference was seen for nLDL (Figure 2B). Uptake studies for 1 hour at 37°C with 50 μg/ml to 200 μg/ml nLDL and oxLDL, respectively, demonstrate a significantly increased uptake of oxLDL in homozygous FH monocytes (Figure 2C). Again there was no difference between the monocytes of heterozygous FH patients and controls. For uptake of nLDL we found no differences between all three groups (Figure 2D). The most prominent difference between FH and control monocytes was observed at 100 μg/ml oxLDL. This concentration was subsequently used to study the time dependent uptake of oxLDL and nLDL for up to 4 hours. At the 4 hour time-point the amounts of both, oxLDL and nLDL, incorporated in monocytes were significantly higher in cells from homozygous FH patients than in those from heterozygous FH patients or from controls (Figure 2E and 2F).

**Table 1:** Baseline characteristics and lipid levels of the study group.

	**homozygous FH**	**heterozygous FH**	**Controls**
**Gender**	female n = 6	female n = 5	female n = 16
	male n = 2	male n = 3	male n = 10

**Age (years)**	30.2 ± 11.1	28.8 ± 7.5	33.8 ± 11.2
**LDL (mg/dl)**	413.82 ± 92.33 (***)	220.64 ± 27.81 (***)	107.03 ± 17.78
**HDL (mg/dl)**	60.33 ± 8.09	54.17 ± 3.71	61.17 ± 6.60
**Triglycerides (mg/dl)**	141.31 ± 39.89	233.61 ± 132.80	107.63 ± 33.04
**Lp(a) (mg/dl)**	676.67 ± 347.06	442.73 ± 420.11	187.53 ± 153.59
**Leukocytes (Gpt/l)**	7.64 ± 0.95	5.45 ± 0.57	6.38 ± 0.8
**Thrombocytes (Gpt/l)**	253.12 ± 22.2	277.39 ± 37.51	258.12 ± 21.97

Values are means ± SD. FH, familial hypercholesterolemia; Gpt, Giga particles; Tpt, Terra particles.

**Table 2:** Plasma concentrations of sterols and sterol to plasma-cholesterol ratio of homozygous and heterozygous FH patients and controls.

	**homozygous FH (n = 5)**		**heterozygous FH (n = 5)**		**Controls (n = 8)**	
	*plasma conc*.	*sterol: Cholesterol*	*plasma conc*.	*sterol: Cholesterol*	*plasma conc*.	*sterol: Cholesterol*
**Cholestanol (mg/dl)**	0.608 ± 0.075 (***)	1,60*10^-3 ^: 1	0.442 ± 0.062 (*)	1,73*10^-3 ^: 1	0.278 ± 0.038	1,59*10^-3 ^: 1
**Lathosterol (mg/dl)**	0.725 ± 0.226	1,78*10^-3 ^: 1	0.563 ± 0.182	1,85*10^-3 ^: 1	0.335 ± 0.121	1,61*10^-3 ^: 1
**Campesterol (mg/dl)**	0.692 ± 0.17 (**)	1,77*10^-3 ^: 1	0.651 ± 0.243 (*)	2.58*10^-3 ^: 1	0.267 ± 0.031	1.40*10^-3 ^: 1
**Campestanol (μg/dl)**	4.773 ± 1.096 (**)	1,23*10^-5 ^: 1	5.237 ± 1.402 (**)	2.09*10^-5 ^: 1	2.151 ± 0.253	1.37*10^-5 ^: 1
**Stigmasterol (μg/dl)**	24.805 ± 7.533 (**)	6.54*10^-5 ^: 1	19.163 ± 6.081 (*)	7.79*10^-5 ^: 1	7.772 ± 1.84	4.25*10^-5 ^: 1
**Sitosterol (mg/dl)**	0.522 ± 0.103 (**)	1.37*10^-3 ^: 1	0.519 ± 0.161 (*)	2.08*10^-3 ^: 1	0.184 ± 0.035	1.06*10^-3 ^: 1
**Sitostanol (μg/dl)**	8.597 ± 2.603 (*)	2.13*10^-5 ^: 1	7.206 ± 2.261 (*)	2.76*10^-5 ^: 1	2.933 ± 0.625	1.96*10^-5 ^: 1
**Brassicosterol (μg/dl)**	84.004 ± 17.864 (**)	2.18*10^-4 ^: 1	80.706 ± 26.213 (*)	3.15*10^-4 ^: 1	29.173 ± 2.612	1.88*10^-4 ^: 1
**Lanosterol (μg/dl)**	39.812 ± 8.347	1.01*10^-4 ^: 1 (**)	34.568 ± 12.712	1.23*10^-4 ^: 1	31.123 ± 5.523	1.79*10^-4 ^: 1
**Desmosterol (mg/dl)**	0.359 ± 0.045 (**)	9.37*10^-4 ^: 1	0.254 ± 0.046	9.54*10^-4 ^: 1	0.167 ± 0.034	9.62*10^-4 ^: 1
**24OH-Cholesterol (ng/ml)**	87.820 ± 18.094 (*)	2.26*10^-7 ^: 1	74.078 ± 9.541	2.90*10^-7 ^: 1	50.585 ± 9.936	3.02*10^-7 ^: 1
**27OH-Cholesterol (ng/ml)**	196.463 ± 27.528 (**)	5.12*10^-7 ^: 1	179.638 ± 19. 015 (*)	6.98*10^-7 ^: 1	49.95 ± 9.533	7.75*10^-7 ^: 1
**7αOH-Cholesterol (ng/ml)**	142.908 ± 49.165 (*)	3.95*10^-7 ^: 1	63.429 ± 10.802	2.43*10^-7 ^: 1	47.363 8.812	3.02*10^-7 ^: 1
**Cholesterol (mg/dl)**	385,748 ± 50.41 (***)		260.55 ± 22.070 (*)		165.669 ± 31.263	

Values are means ± SD. FH, familial hypercholesterolemia.

**Figure 3:**
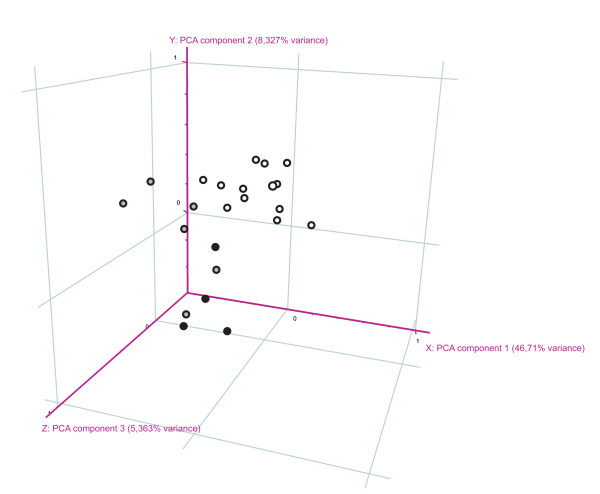
**Principal component analysis (PCA) of gene expression profiles from monocytes**. PCA of 35,264 transcripts expressed in monocytes of homozygous FH patients (filled circles), heterozygous FH patients (grey filled circles) and controls (open circles), respectively, that passed pre-filter criteria (described in Material and Methods). Gene signatures in monocytes from FH patients are sufficient to distinguish between FH and healthy controls.

As FH monocytes show a clearly increased uptake of LDL and concomitantly FH carriers have significantly increased serum oxLDL levels, we analyzed the cellular response to the higher uptake of cholesterol in FH monocytes using microarray analysis (quality control data [see Additional file 2]). The transcriptomes of freshly isolated monocytes from FH patients (baseline characteristics are given in Table 1) were analyzed using Affymetrix HG133 Plus 2.0 microarrays. 35,264 transcripts in monocytes that fulfilled the threshold criteria for expressed genes (a raw expression value of at least 20 in half of all measured array was considered as "expressed") were analyzed by principal component analysis (PCA). Gene expression profiles of monocytes from FH patients and from controls were clearly separated from each other (Figure 3). A Welch T test with correction for multiple testing (p < 0.05; Benjamini Hochberg correction, False Discovery Rate = 0.05) showed that 2,318 genes in monocytes were differently expressed. Of these genes 1,617 were up-regulated and 701 were down-regulated in monocytes of FH patients. Using DAVID 2008, a web based tool for functional annotation clustering [9], we identified among regulated transcripts functional clusters of genes coding for proteins involved in 1) intracellular protein transport, 2) clathrin coated vesicle transport, 3) lysosome and lytic vacuole, 4) regulation of JNK activity and 5) endocytosis (Table 3). These data and the observed increased uptake of nLDL and oxLDL by FH monocytes point to differences in LDL uptake that could involve scavenger receptors, as a role for CD36 and LRP1 has been reported for oxLDL and nLDL uptake in macrophages, respectively. Further, a pathway analysis of the differentially expressed genes showed that the SAPK-JNK, p38 and MAPK pathways belong to the most prominently regulated signaling pathways in FH monocytes. Recent publications have shown that these signaling pathways are crucial for the development of atherosclerosis and are regulated by oxLDL in a scavenger receptor dependent manner [13,14]. Also among the top 5 regulated signaling pathways are the apoptosis and the death receptor pathways, which are both down-regulated. Our data thus support recently published work from Namgaladze et al. who demonstrated that oxLDL can attenuate apoptosis in monocytes [15]. Further, also the VEGF signalling pathway is up-regulated in FH monocytes. Interestingly there is a recent publication reporting that C-reactive protein (CRP), a suggested independent risk marker of atherosclerosis, induces VEGF expression via Erk/PI3K signalling. Expression of these signalling pathways is also increased in FH monocytes [16].

**Table 3:** Functional cluster analysis of differentially regulated genes in monocytes between FH individuals and controls.

Welch T-Test (p < 0.05, Benjamini Hochberg Testing Correction FDR = 0.05)	Functional GO cluster	Genes	Group Enrichment Score
2,318 transcripts regulated	protein transport, establishment of protein localization, intracellular protein transport	130	7.62
	clathrin coated vesicle, trans Golgi network transport vesicle, Golgi associated vesicle membrane, transport vesicle membrane	15	2.86
	vacuole, lysosome, lytic vacuole	37	2.42
	regulation of JNK activity activation of JNK activity,, positive activation of JNK activity, activation of MAPK activity	14	2.32
	clathrin coated endocytotic vesicle, AP-2 adaptor complex, endocytotic vesicle membrane	4	2.00

**Table 4:** Individual LDL-R characteristics of each FH patient those monocytes where analyzed by microarray.

homozygous FH	Mutation
Patient 1	C88R (FH Münster 1), D333G (FH Münster 2); < 5% LDL-R activity, binding defect
Patient 2	W556R; < 5% receptor activity (class 2A)
Patient 3	W556R; < 5% receptor activity (class 2A)
Patient 4	W556R; < 5% receptor activity (class 2A)
Patient 5	LDL-R binding defect demonstrated by fibroblast LDL binding assay
Patient 6–8	Diagnosis based on clinical parameters according to the Simon Broome Register Group criteria (xanthomas, lipid profile, familial history)
heterozygous FH	

Patient 9	promoter defect -135 bp C->G, 5–15% activity
Patient 10	C88R (FH Münster 1), 15–30% receptor activity binding defect
Patient 11	D333G (FH Münster 2), 15–30% receptor activity binding defect
Patient 12	C88R (FH Münster 1), 15–30% receptor activity binding defect
Patient 13	LDL-R binding defect demonstrated by fibroblast LDL binding assay
Patient 14	W556R; < 5% receptor activity (class 2A)
Patient 15	insertion of G at 588 bp -> STOP at codon 178
Patients 16	insertion of G at 588 bp -> STOP at codon 178

**Figure 4:**
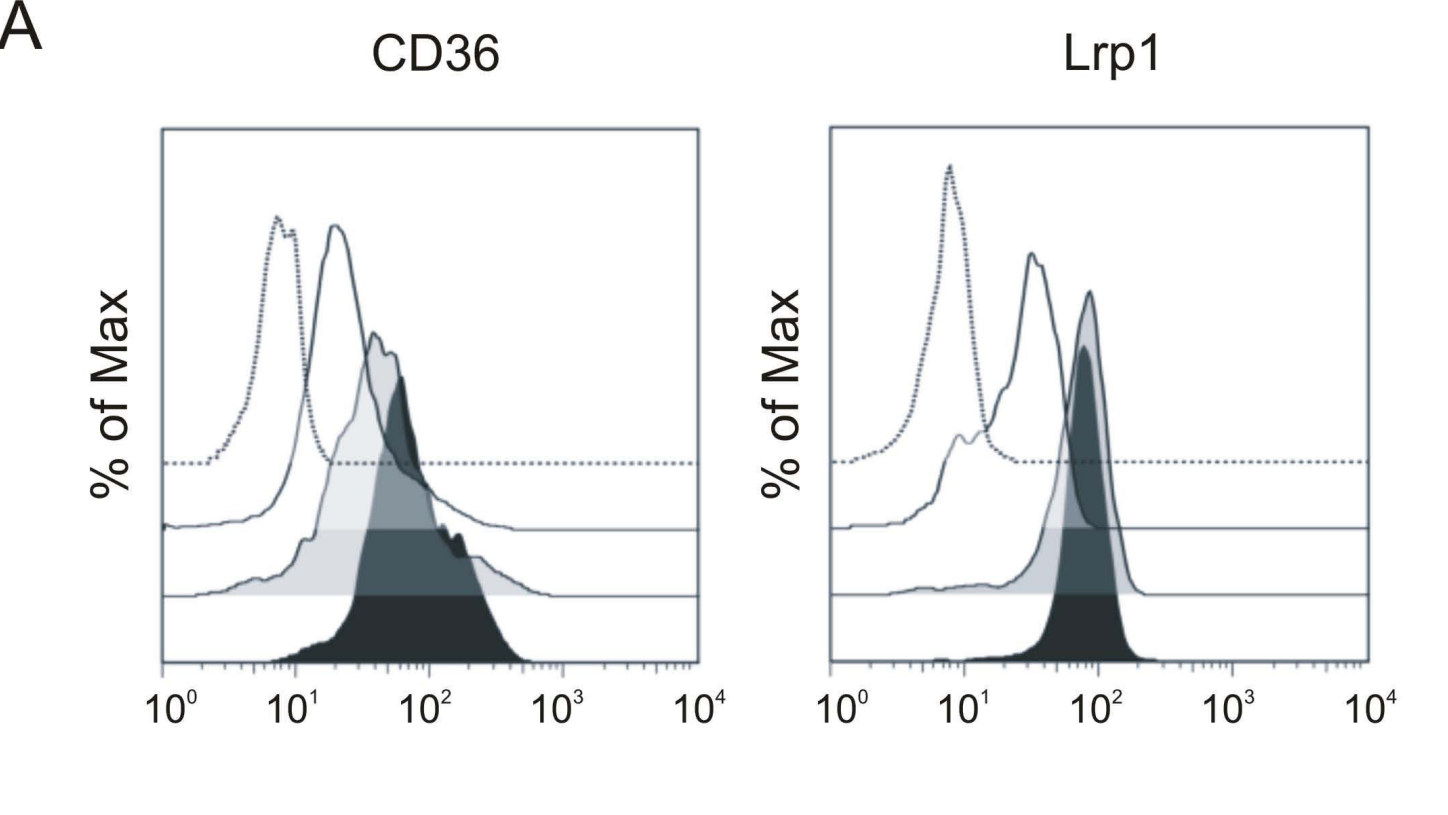
**Increased CD36 and CD91/Lrp1 levels on the surface of monocytes from FH patients and their role in LDL uptake**. *A*: Representative measurements by flow cytometry of the cell surface expression of CD36 and CD91/Lrp1 on monocytes of a control (no filling), of a heterozygous FH patient (gray filling) and of a homozygous FH patient (black filling). One representative measurement of an antibody isotype control is illustrated as a black dotted line. The y-axis shows the percentage of maximum events. *B*: Western Blot analysis of CD91/Lrp1 of four homozygous FH patients and four controls. β-actin was used as loading control. *C*: Monocytes of homozygous FH patients (black filled bars), heterozygous FH patients (grey filled bars) and control individuals (open bars) were incubated with CD36-blocking antibody FA6-152, control murine IgG or left untreated for 0,5 h at 37°C; after incubation cells were incubated with 100 μg/ml AF647 labelled oxLDL for 4 h at 37°C. (homozygous FH n = 5,, heterozygous FH patients n = 4, healthy controls n = 5) *D*: Monocytes of homozygous FH patients (black filled bars), heterozygous FH patients (grey filled bars) and control individuals (open bars) were incubated with CD91/Lrp1-blocking antibody, control rabbit IgG or left untreated for 0,5 h at 37°C; after incubation cells were incubated with 100 μg/ml AF647 labelled nLDL for 4 h at 37°C. Experiments in C and D were done in duplicate. Histogram bars indicate the mean ± SD (** p < 0.01, *** p < 0.001).

An analysis of the gene expression profiles of FH monocytes revealed an up-regulation of CD36 and LRP1 which was confirmed by qRT-PCR [see Additional file 3]. Using flow cytometry, we demonstrated that also the protein expression of both receptors was increased in FH monocytes (Figure 4A). The elevated protein expression of Lrp1 in homozygous FH monocytes was also verified by Western Blot (Figure 4B [see Additional File 4]). CD36 which plays a major role for the acquisition of excess cellular cholesterol in tissue macrophages in mice [17] was tested for its contribution to the increased uptake of oxLDL and nLDL by FH monocytes. Using a CD36 blocking antibody we found a reduction of oxLDL uptake in homozygous as well as heterozygous FH monocytes by more than 50 percent (Figure 4C [see Additional file 4]). OxLDL uptake was also reduced in monocytes of healthy controls by blocking CD36. However, monocytes of FH patients took up much more oxLDL via CD36 compared to healthy controls. In contrast to oxLDL uptake, a role of CD36 in the uptake of nLDL was excluded (data not shown). To test whether Lrp1 has a function in nLDL uptake of monocytes we performed experiments with a blocking antibody against Lrp1. As shown in Figure 4D [see Additional file 4] Lrp1 blocking did not result in any change of the uptake of nLDL in either FH or control monocytes.

**Figure 5:**
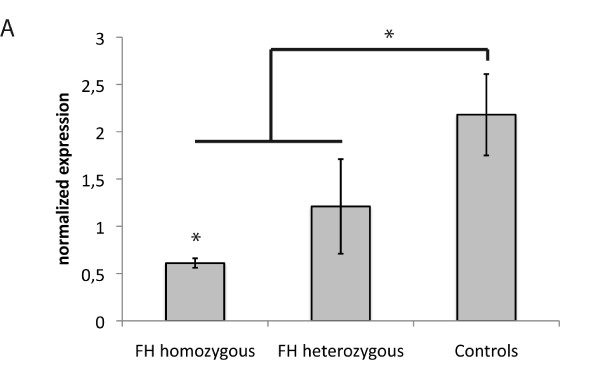
**Expression of NPC1 and ABCA1 in monocytes of FH patients and of healthy control individuals**. *A*: qRT-PCR shows that mRNA expression of NPC1 is decreased in FH monocytes. *B*: Western Blot analysis of Npc1 and Abca1 in monocytes of three homozygous FH patients and three controls. C: qRT-PCR analysis of ABCA1 expression fold change during differentiation of monocytes to macrophages over 10 days of homozygous FH patients (n = 4) and healthy controls (n = 4). Histogram bars indicate the mean ± SD. Expression levels in control monocytes were compared with those of all FH carriers and with FH homozygotes only (* p < 0.05, ** p < 0.01).

Npc1 and Abca1 have been identified as crucial for the distribution and secretion of cholesterol within macrophages [18,19]. We therefore analyzed the expression of these two genes in FH monocytes and found the expression of both, NPC1 and ABCA1 mRNAs down-regulated in FH on microarrays. Using qRT-PCR we verified the reduced expression of NPC1 (Figure 5A). Also the protein expression of NPC1 and ABCA1 was found reduced in homozygous FH (Figure 5B [see Additional file 5]). This was further verified in monocytes differentiated to macrophages in cell culture using GAPDH as an internal standard. Although ABCA1 mRNA concentration increased during the differentiation process the relative fold change of ABCA1 expression is markedly smaller in monocyte derived macrophages of homozygous FH patients compared to those from control subjects (Figure 5 [see Additional file 5]). It is thus likely that the lipid overload of FH monocytes observed by Oil Red O staining results from both, an increased oxLDL and nLDL uptake and a reduced cellular efflux.

**Figure 6:**
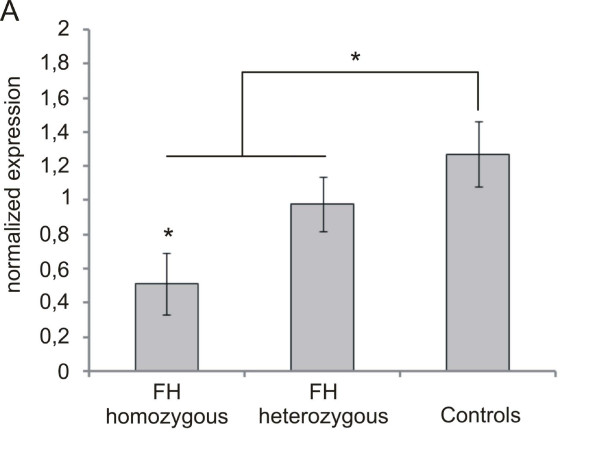
**Analyses of gene and protein expression by qRT-PCR and Western Blot reveal down-regulation of members of the SREBP-pathway**. *A*: INSIG2. *B*: SREBF2. *C*: Scap.*D*: HMGCR. β-actin was used as loading control. Histogram bars indicate the mean ± SD (* p < 0.05, ** p < 0.01).

We also measured the expression of genes responsible for cholesterol *de-novo *synthesis of the SREBP pathway, which regulates the cellular cholesterol content. The analysis of gene expression profiles revealed that most SREBP pathway genes were down-regulated in FH monocytes. In addition, both, quantitative real-time PCR (qRT-PCR) and Western blotting exhibited a significantly reduced expression of INSIG2, SREBF2 and SCAP (Figure 6A and Figure 6B, C [see Additional file 6]). Moreover, the gene for the key regulating enzyme of cholesterol *de novo *synthesis, HMGCR, and the LDLR were expressed at lower levels in FH than in control monocytes (Figure 6D [see Additional file 6] and data not shown). The SREBP pathway is also down regulated in monocytes from heterozygous FH patients. As in both, FH monocytes and control monocytes LDL uptake increases with rising concentrations of LDL in the cell culture, we assume that this concentration dependence in LDL uptake also occurs *in vivo*. Hence, monocytes freshly isolated from heterozygous FH patients, who have significantly elevated serum LDL levels, have likely taken up more LDL during circulation which is expected to result in a down regulation of cholesterol sensitive SREBP pathway genes. The observed down-regulation of the SREBP pathway is not expected to be caused by medication as both, FH patients receiving medication as well as FH patients without medication, show a similar reduction in SREBP gene expression [see Additional file 7].

We also found the intracellular levels of lanosterol, a precursor in the *de-novo *cholesterol biosynthesis, reduced by about 50% in monocytes from homozygous FH patients (control monocytes: 0.019887 μg lanosterol/mg cellular dry weight; FH monocytes: 0.0100980 μg lanosterol/mg cellular dry weight; p < 0.05). Taken together, these data argue for a down-regulation of the SREBP-pathway due to an elevated uptake of nLDL by an unknown mechanism and oxLDL via CD36 resulting in a cellular cholesterol overload of FH monocytes.

## Conclusion

An important role of macrophages in arterial wall metabolism and in the pathogenesis of atherosclerosis is well documented [1]. We were interested to see, if already their precursors in the bloodstream, the monocytes possess an increased lipid content. Oil Red O staining of freshly isolated monocytes revealed that FH monocytes have higher lipid content than control monocytes. They also show an increased uptake of nLDL and oxLDL. This and the three to fourfold higher plasma concentrations of oxLDL and nLDL in FH patients can explain the observed higher number of lipid-laden monocytes in FH. Our data is in agreement with recent reports from LDLR deficient mice which have an increased intracellular cholesterol content in macrophages [20].

To assess the consequences of the intracellular lipid enrichment *in vivo *we analyzed gene expression profiles from freshly isolated FH patient monocytes and from control monocytes using whole genome cRNA microarrays. The gene expression profiles of FH monocytes were clearly distinct from those of healthy individuals as demonstrated by unsupervised principal component analysis. Further, GO enrichment analyses pointed to an altered vesicle transport from the plasma membrane to the lysosome. We identified two scavenger receptors, CD36 and Lrp1 to be overexpressed in FH monocytes. CD36 has been identified as one of the principal receptors for oxLDL uptake by macrophages in mice [17,21]. The expression of this receptor is largely restricted to lymphoid and haematopoietic lineages, including monocytes, macrophages, platelets and endothelial cells [22]. Blocking of CD36 uptake with an antibody resulted in a reduction of oxLDL uptake by more than 50% which identified CD36 as the major receptor for oxLDL in circulating monocytes from FH patients and healthy controls. However, the total amount of oxLDL uptake via CD36 clearly distinguishes FH monocytes from control monocytes.

Although none of the homozygous FH patients who took part in this study had a residual LDL-R activity of more than 5 percent and LDL-R expression was slightly down-regulated in these individuals (data not shown), we observed a 50% higher nLDL uptake in FH monocytes. This finding was surprising, as LDLR defective fibroblasts do not take up nLDL. It indicates that FH monocytes have an alternative uptake mechanism for nLDL not present in FH fibroblasts. Although Lrp1 was shown to be a receptor for the binding and uptake of native LDL in peritoneal and J774 macrophages [23,24], we found no participation of this receptor in the uptake of nLDL by FH monocytes. Llorente-Cortes et al. recently reported that in monocyte-derived macrophages Lrp1-expression is regulated by Srebf2, whereupon a down-regulation of Srebf2, due to elevated intracellular cholesterol, results in a down-regulation of the LDL receptor and in an up-regulation of Lrp1 which is in good concordance with our findings [25,26]. Although we cannot explain the mechanism for nLDL uptake, macropinocytosis could be one possible explanation, as it has recently been shown that this process contributes to foam cell formation by the uptake of nLDL in macrophages [27].

To prevent a cholesterol/lipid overload macrophages are able to release excessive cholesterol via Npc1 mediated transport to intracellular destinations and the subsequent efflux of cholesterol to apo A1 via Abca1 [3]. Both proteins were found down-regulated in FH monocytes, which further contributes to the lipid overload of FH monocytes. Studies regarding the function of oxLDL with respect to ABCA1 transcription have indicated that in macrophages oxysterols lead to the induction of ABCA1-expression via LXR [28,29] which is different from our observation in FH monocytes where expression of LXRα and LXRβ is unchanged. However, Zhou et al recently reported that LDLR deficiency impairs ABCA1 expression in macrophages via a SREBP dependent mechanism [20]. ABCA1 expression was also inhibited under oxysterol treatment and its regulation was independent from LXRα and LXRβ expression. They reported that LDLR is crucial in the regulation of ABCA1 expression by inhibiting SREBP1 proteolysis. Lack of LDLR led to abnormal SREBP response which resulted in both, reduced ABCA1 expression and cholesterol efflux [20]. This may explain the reduced ABCA1 expression observed in FH monocytes. Our observation of an impaired ABCA1 gene expression in response to human LDL receptor deficiency during differentiation of FH monocytes into macrophages suggests that deranged reverse cholesterol transport is a major contributor to foam cell formation in FH. Furthermore, the lipid overload of FH monocytes is likely to cause the observed down-regulation of the cholesterol sensitive SREBP2, which is a positive regulator of ABCA1 transcription [30] and thus can aid to further enhance ABCA1 repression. It has been reported that ABCA1 is absent in cells of atherosclerotic lesions [31], which demonstrates that the tight regulation of this efflux system is critical to atherosclerosis development. Interestingly, INSIG2 and SCAP, which are also members of the SREBP pathway that regulates intracellular cholesterol content [32], and the rate limiting enzyme of the cholesterol *de novo *synthesis, HMGCR, are down-regulated in FH monocytes as well. In addition, intracellular lanosterol, a precursor of cholesterol in the *de novo *biosynthesis pathway, was found to be decreased in FH monocytes, further arguing for a cellular lipid overload caused by elevated lipid influx and reduced efflux.

In conclusion, we have demonstrated significant differences between circulating monocytes from patients with FH and those from control persons. FH monocytes contain larger amounts of lipids as evidenced by Oil Red O staining. This finding can be explained by the observed increased uptake of nLDL and oxLDL, which is accompanied by a down-regulation of the SREBP pathway and its target genes, and by a reduced level of intracellular lanosterol. Moreover, a down-regulation of key proteins of cholesterol transport and cholesterol efflux was observed. The increased uptake of oxLDL into FH monocytes was found to be largely mediated by the scavenger receptor CD36. We have identified processes that substantially alter the metabolism of circulating monocytes in an extreme hypercholesterolemic environment such as the blood compartment in FH. These changes that lead to a lipid overload of monocytes already in the circulation may promote the formation of local inflammatory sites that favour the onset of atherosclerosis. The large numbers of genes that are significantly altered in FH monocytes suggest, however, that many as yet unidentified genes also contribute to this process.

## Competing interests

The authors declare that they have no competing interests.

## Authors' contributions

SM designed the study, SM and KR carried out most of the experiments and prepared the manuscript; PB participated on microarray measurement and statistical analysis; SK performed isolation of LDL and participated in manuscript preparation and data interpretation; DL carried out mass spectrometry measurements; MS performed molecular analysis of LDLR defects; RH participated on preparing the manuscript and on data interpretation; HF participated on the preparation of the manuscript.

## Pre-publication history

The pre-publication history for this paper can be accessed here:



## Supplementary Material

Additional file 1 **Serum oxLDL levels and neutral lipid content of monocytes from FH patients and healthy individuals.** The data provides show an increased lipid content in monocytes of FH patients compared to healthy individuals. Click here for file

Additional file 2 **Quality control of microarry raw data.** A: legend of array index. B: Whisker box plots of log2 transformed microarray raw data. The centers of the boxes represent the median of the genes, upper and lower ends of the boxes represent the 75^th ^and 25^th ^quartiles, respectively. Upper whisker shows the 90^th^, the lower whisker the 10^th ^percentile. C: Density plot of log2 transformed raw data. D: Plot of 3'/5' GAPDH and beta-actin ratios. Numbers behind the sample names represent % of present calls and average background, respectively. The blue line indicates the scaling factor. E: Array vs. array intensity correlation plot. Click here for file

Additional file 3 **Analysis of gene expression by qRT-PCR for CD36 and LRP1.** mRNA expression of A: CD36 and B: LRP1 is elevated in monocytes of patients with homozygous or heterozygous FH compared to controls. Histogram bars indicate the mean ± SD (* p < 0.05, ** p < 0.01). Click here for file

Additional file 4 **Increased CD36 and CD91/Lrp1 levels on the surface of monocytes from FH patients and their role in LDL uptake.** Figure 4A–D in this file shows increased expression of CD36 and CD91/Lrp1 on monocytes of FH patients. CD36 mediates uptake of oxLDL in monocytes of FH patients and healthy individuals. CD91/Lrp1 is not involved in nLDL uptake. Click here for file

Additional file 5 **Expression of NPC1 and ABCA1 in monocytes of FH patients and of healthy control individuals.** The data provided show that NPC1 as well as ABCA1 expression is deceased in FH monocytes. ABCA1 expression is further decreased during monocyte to macrophage differentiation of FH monocytes compared to monocytes of healthy individuals. Click here for file

Additional file 6 **Analyses of gene and protein expression by qRT-PCR and Western Blot reveal down-regulation of members of the SREBP-pathway.** Genes and proteins of the SREBP pathway are down-regulated in monocytes of FH patients. Click here for file

Additional file 7 **qRT-PCR analysis of SREBP gene expression of FH patients receiving statin medication and FH patients without statin therapy.** Statin therapy has no effect of SREBP gene expression in monocytes. Histogram bars indicate the mean ± SD Click here for file

## References

[B1] Lusis AJ (2000). Atherosclerosis. Nature.

[B2] Kong WJ, Liu J, Jiang JD (2006). Human low-density lipoprotein receptor gene and its regulation. J Mol Med.

[B3] Boadu E, Francis GA (2006). The role of vesicular transport in ABCA1-dependent lipid efflux and its connection with NPC pathways. J Mol Med.

[B4] van Eck M, Pennings M, Hoekstra M, Out R, van Berkel TJ (2005). Scavenger receptor BI and ATP-binding cassette transporter A1 in reverse cholesterol transport and atherosclerosis. Curr Opin Lipidol.

[B5] van Berkel TJ, Out R, Hoekstra M, Kuiper J, Biessen E, van Eck M (2005). Scavenger receptors: friend or foe in atherosclerosis?. Curr Opin Lipidol.

[B6] Tacke F, Randolph GJ (2006). Migratory fate and differentiation of blood monocyte subsets. Immunobiology.

[B7] van Aalst-Cohen ES, Jansen AC, de Jongh S, de Sauvage Nolting PR, Kastelein JJ (2004). Clinical, diagnostic, and therapeutic aspects of familial hypercholesterolemia. Semin Vasc Med.

[B8] Irizarry RA, Hobbs B, Collin F, Beazer-Barclay YD, Antonellis KJ, Scherf U, Speed TP (2003). Exploration, normalization, and summaries of high density oligonucleotide array probe level data. Biostat.

[B9] Dennis G, Sherman BT, Hosack DA, Yang J, Gao W, Lane HC, Lempicki RA (2003). DAVID: Database for Annotation, Visualization, and Integrated Discovery. Genome Biol.

[B10] Hosack DA, Dennis G, Sherman BT, Lane HC, Lempicki RA (2003). Identifying biological themes within lists of genes with EASE. Genome Biol.

[B11] Havel RJ, Eder HA, Bragdon JH (1955). The distribution and chemical composition of ultracentrifugally separated lipoproteins in human serum. J Clin Invest.

[B12] Teunissen CE, De Vente J, von Bergmann K, Bosma H, van Boxtel MPJ, De Bruijn C, Jolles J, Steinbusch HWM, Lutjohann D (2003). Serum cholesterol, precursors and metabolites and cognitive performance in an aging population. Neurobiology of Aging.

[B13] Katayama I, Hotokezaka Y, Matsuyama T, Sumi T, Nakamura T (2008). Ionizing radiation induces macrophage foam cell formation and aggregation through JNK-dependent activation of CD36 scavenger receptors. Int J Radiat Oncol Biol Phys.

[B14] Lei ZB, Zhang Z, Jing Q, Qin YW, Pei G, Cao BZ, Li XY (2002). OxLDL upregulates CXCR2 expression in monocytes via scavenger receptors and activation of p38 mitogen-activated protein kinase. Cardiovasc Res.

[B15] Namgaladze D, Kollas A, Brune B (2008). Oxidized LDL attenuates apoptosis in monocytic cells by activating ERK signaling. J Lipid Res.

[B16] Bello G, Cailotto F, Hanriot D, Kolopp-Sarda M-N, Latger-Cannard V, Hess K, Zannad F, Longrois D, Ropars A (2008). C-reactive protein (CRP) increases VEGF-A expression in monocytic cells via a PI3-kinase and ERK 1/2 signaling dependent pathway. Atherosclerosis.

[B17] Podrez EA, Febbraio M, Sheibani N, Schmitt D, Silverstein RL, Hajjar DP, Cohen PA, Frazier WA, Hoff HF, Hazen SL (2000). Macrophage scavenger receptor CD36 is the major receptor for LDL modified by monocyte-generated reactive nitrogen species. J Clin Invest.

[B18] Reid PC, Sugii S, Chang TY (2003). Trafficking defects in endogenously synthesized cholesterol in fibroblasts, macrophages, hepatocytes, and glial cells from Niemann-Pick type C1 mice. J Lipid Res.

[B19] Wang N, Silver DL, Thiele C, Tall AR (2001). ATP-binding cassette transporter A1 (ABCA1) functions as a cholesterol efflux regulatory protein. J Biol Chem.

[B20] Zhou X, He W, Huang Z, Gotto AM, Hajjar DP, Han J (2008). Genetic Deletion of Low Density Lipoprotein Receptor Impairs Sterol-induced Mouse Macrophage ABCA1 Expression: A NEW SREBP1-DEPENDENT MECHANISM. J Biol Chem.

[B21] Kunjathoor VV, Febbraio M, Podrez EA, Moore KJ, Andersson L, Koehn S, Rhee JS, Silverstein R, Hoff HF, Freeman MW (2002). Scavenger receptors class A-I/II and CD36 are the principal receptors responsible for the uptake of modified low density lipoprotein leading to lipid loading in macrophages. J Biol Chem.

[B22] Calvo D, Dopazo J, Vega MA (1995). The CD36, CLA-1 (CD36L1), and LIMPII (CD36L2) gene family: cellular distribution, chromosomal location, and genetic evolution. Genomics.

[B23] Sakr SW, Eddy RJ, Barth H, Wang F, Greenberg S, Maxfield FR, Tabas I (2001). The uptake and degradation of matrix-bound lipoproteins by macrophages require an intact actin Cytoskeleton, Rho family GTPases, and myosin ATPase activity. J Biol Chem.

[B24] Wu SM, Pizzo SV (1996). Low-density lipoprotein receptor-related protein/alpha 2-macroglobulin receptor on murine peritoneal macrophages mediates the binding and catabolism of low-density lipoprotein. Arch Biochem Biophys.

[B25] Llorente-Cortes V, Costales P, Bernues J, Camino-Lopez S, Badimon L (2006). Sterol Regulatory Element-binding Protein-2 Negatively Regulates Low Density Lipoprotein Receptor-related Protein Transcription. J Mol Biol.

[B26] Llorente-Cortes V, Royo T, Otero-Vinas M, Berrozpe M, Badimon L (2007). Sterol regulatory element binding proteins downregulate LDL receptor-related protein (LRP1) expression and LRP1-mediated aggregated LDL uptake by human macrophages. Cardiovasc Res.

[B27] Kruth HS, Jones NL, Huang W, Zhao B, Ishii I, Chang J, Combs CA, Malide D, Zhang WY (2005). Macropinocytosis is the endocytic pathway that mediates macrophage foam cell formation with native low density lipoprotein. J Biol Chem.

[B28] Chawla A, Boisvert WA, Lee CH, Laffitte BA, Barak Y, Joseph SB, Liao D, Nagy L, Edwards PA, Curtiss LK (2001). A PPAR gamma-LXR-ABCA1 pathway in macrophages is involved in cholesterol efflux and atherogenesis. Mol Cell.

[B29] Costet P, Luo Y, Wang N, Tall AR (2000). Sterol-dependent transactivation of the ABC1 promoter by the liver X receptor/retinoid X receptor. J Biol Chem.

[B30] Wong J, Quinn CM, Brown AJ (2006). SREBP-2 positively regulates transcription of the cholesterol efflux gene, ABCA1, by generating oxysterol ligands for LXR. Biochem J.

[B31] Forcheron F, Legedz L, Chinetti G, Feugier P, Letexier D, Bricca G, Beylot M (2005). Genes of cholesterol metabolism in human atheroma: overexpression of perilipin and genes promoting cholesterol storage and repression of ABCA1 expression. Arterioscler Thromb Vasc Biol.

[B32] Brown MS, Goldstein JL (1997). The SREBP pathway: regulation of cholesterol metabolism by proteolysis of a membrane-bound transcription factor. Cell.

