# Supplementary figures and images for: Monocytes of patients with familial hypercholesterolemia show alterations in cholesterol metabolism
Source: BMC Med Genomics. 2008 Nov 28;1:60. doi: 10.1186/1755-8794-1-60 (PMC2633353; doi:10.1186/1755-8794-1-60)

A

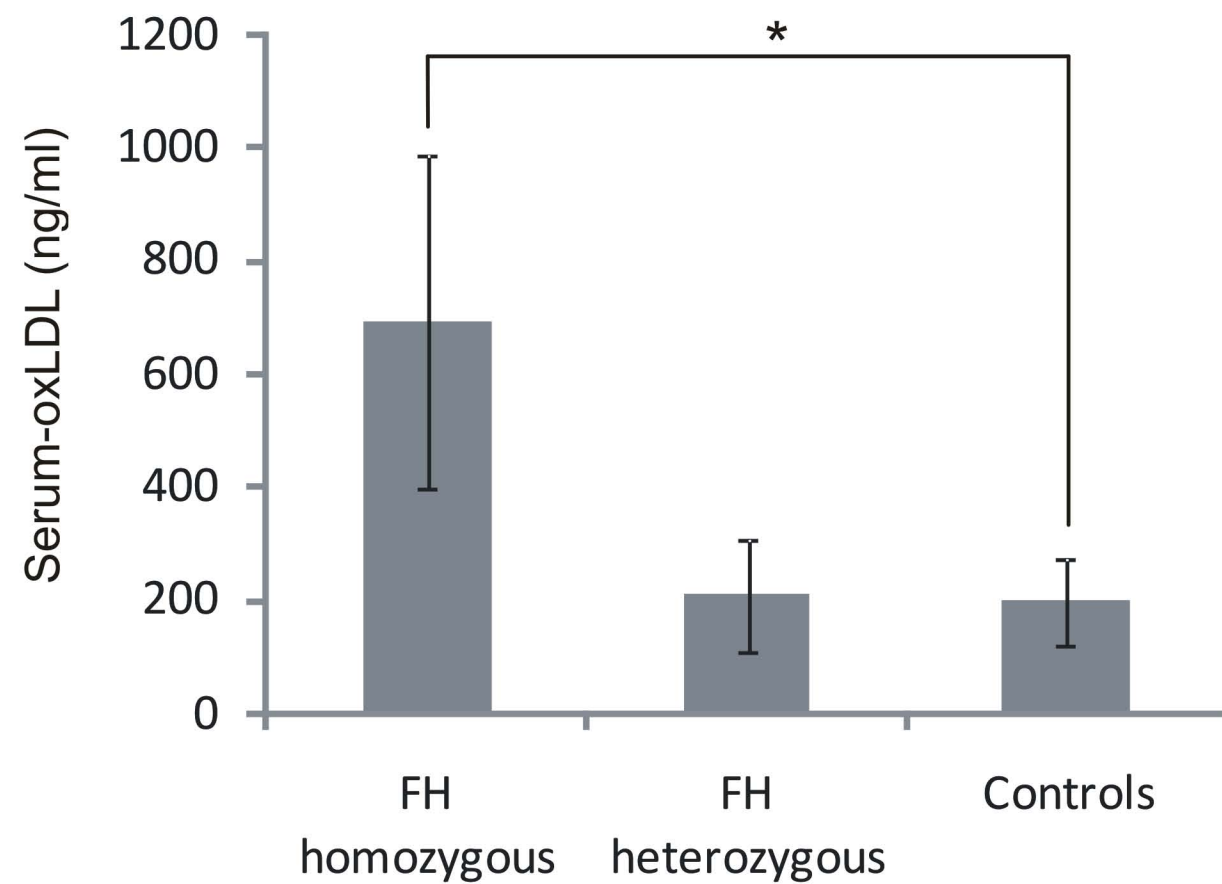

B

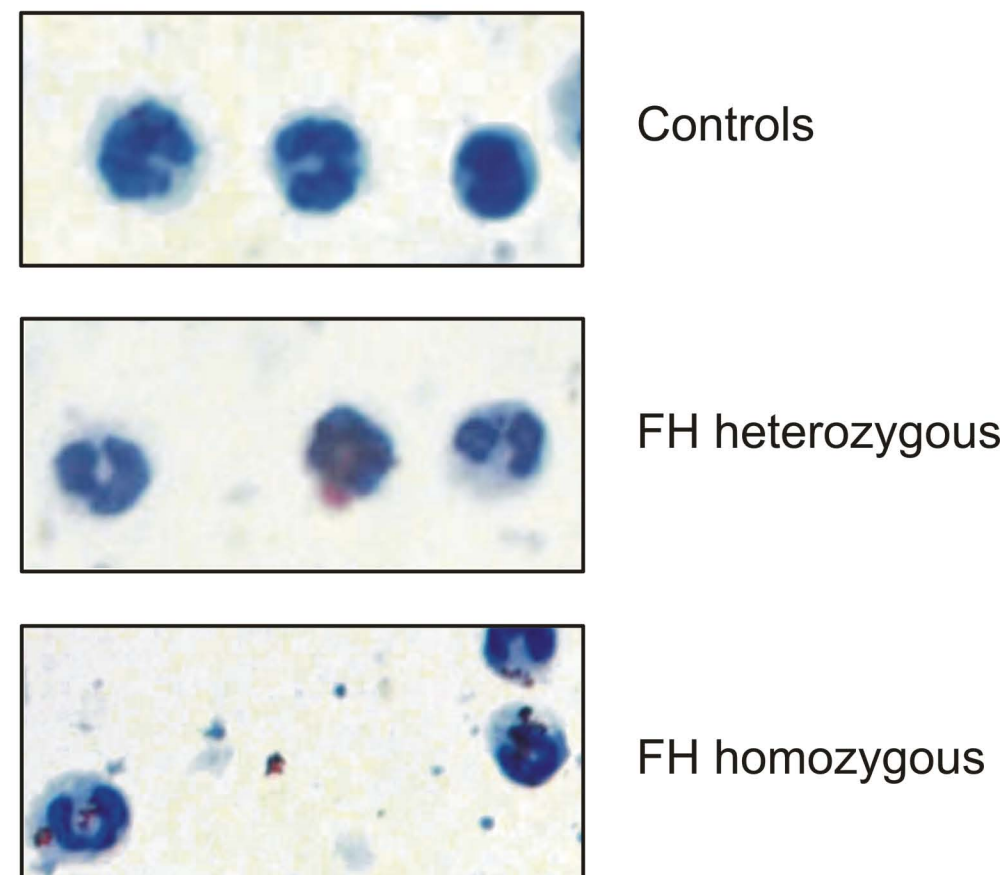

C

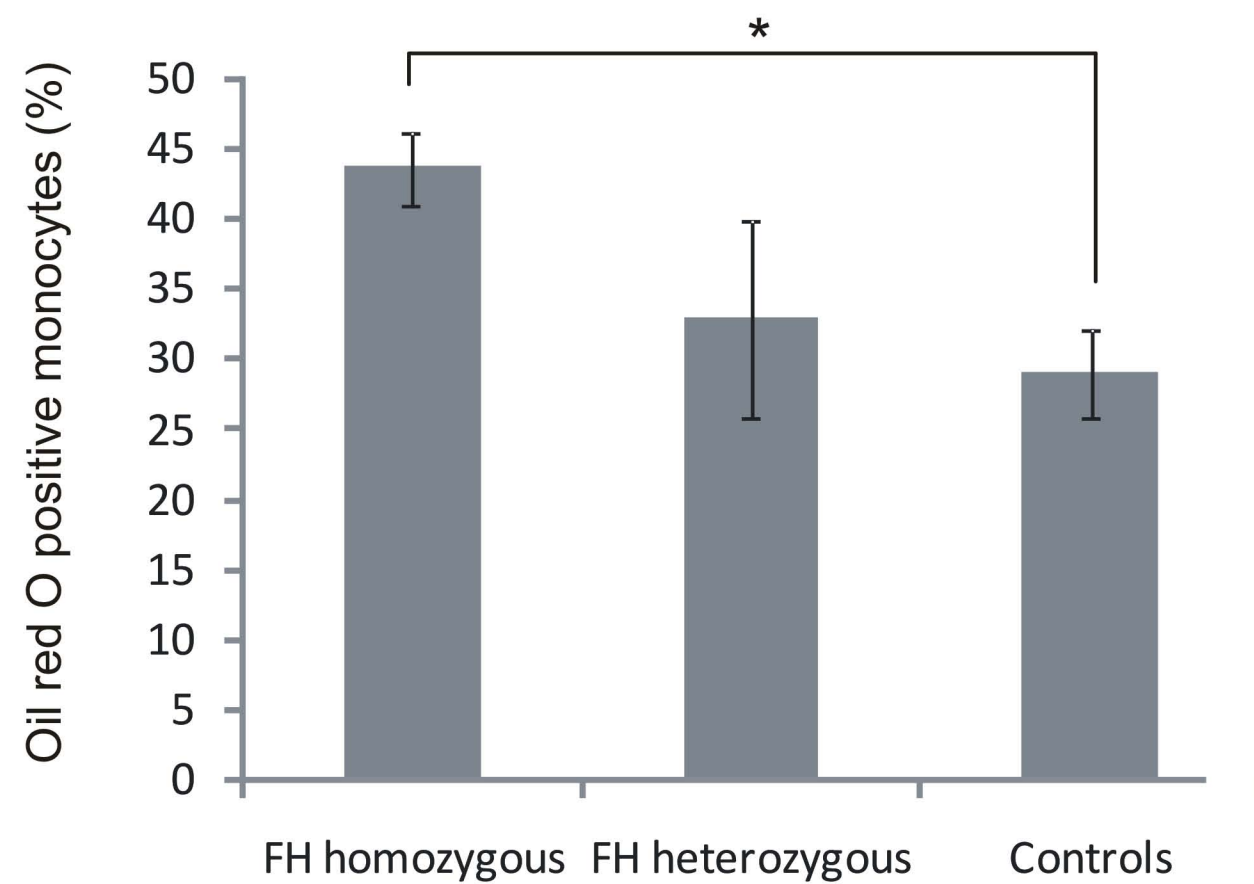

Supplement: Additional file 1 — Serum oxLDL levels and neutral lipid content of monocytes from FH patients and healthy individuals. The data provides show an increased lipid content in monocytes of FH patients compared to healthy individuals. [file 1755-8794-1-60-S1.pdf]

**A**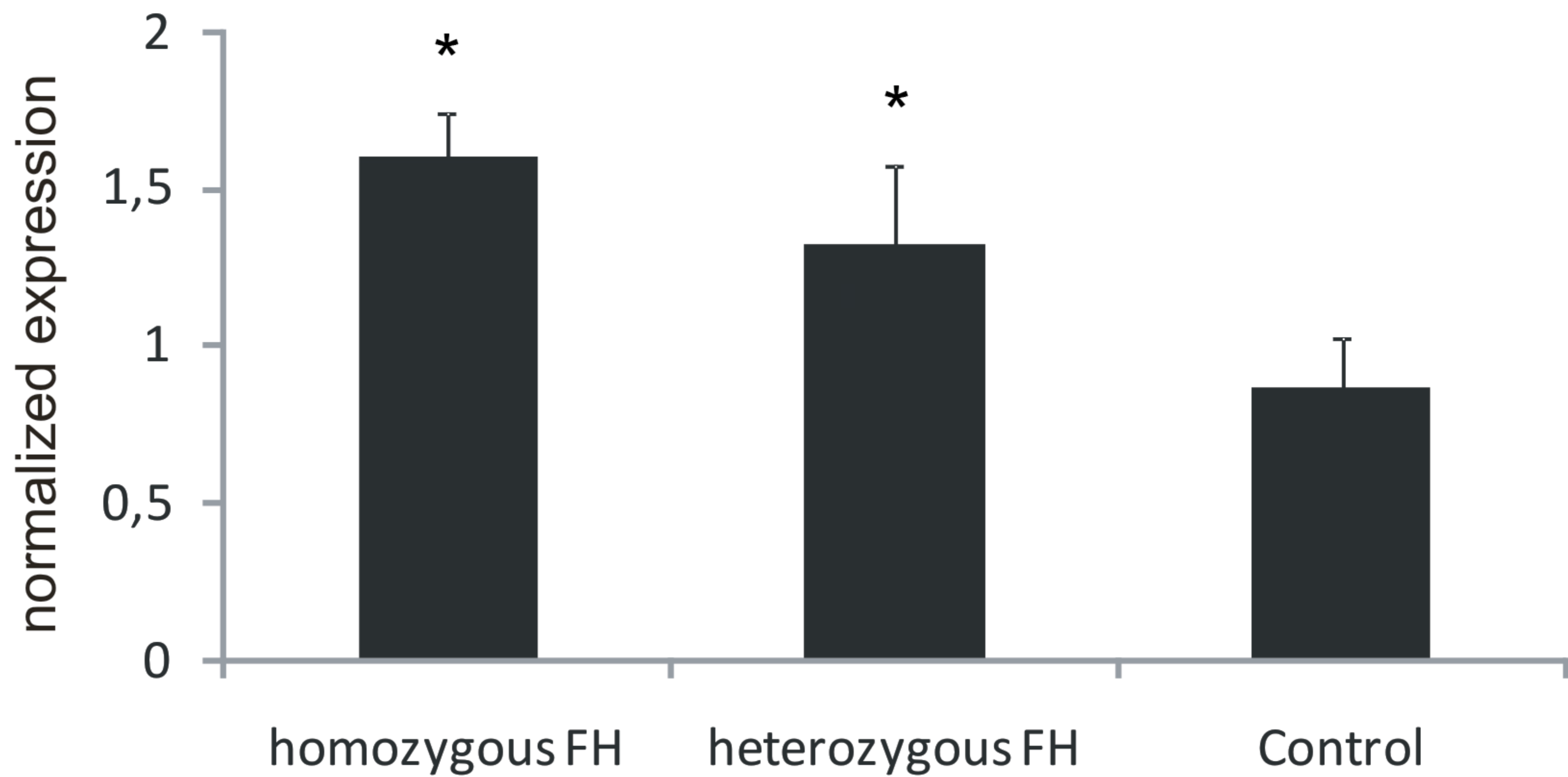**B**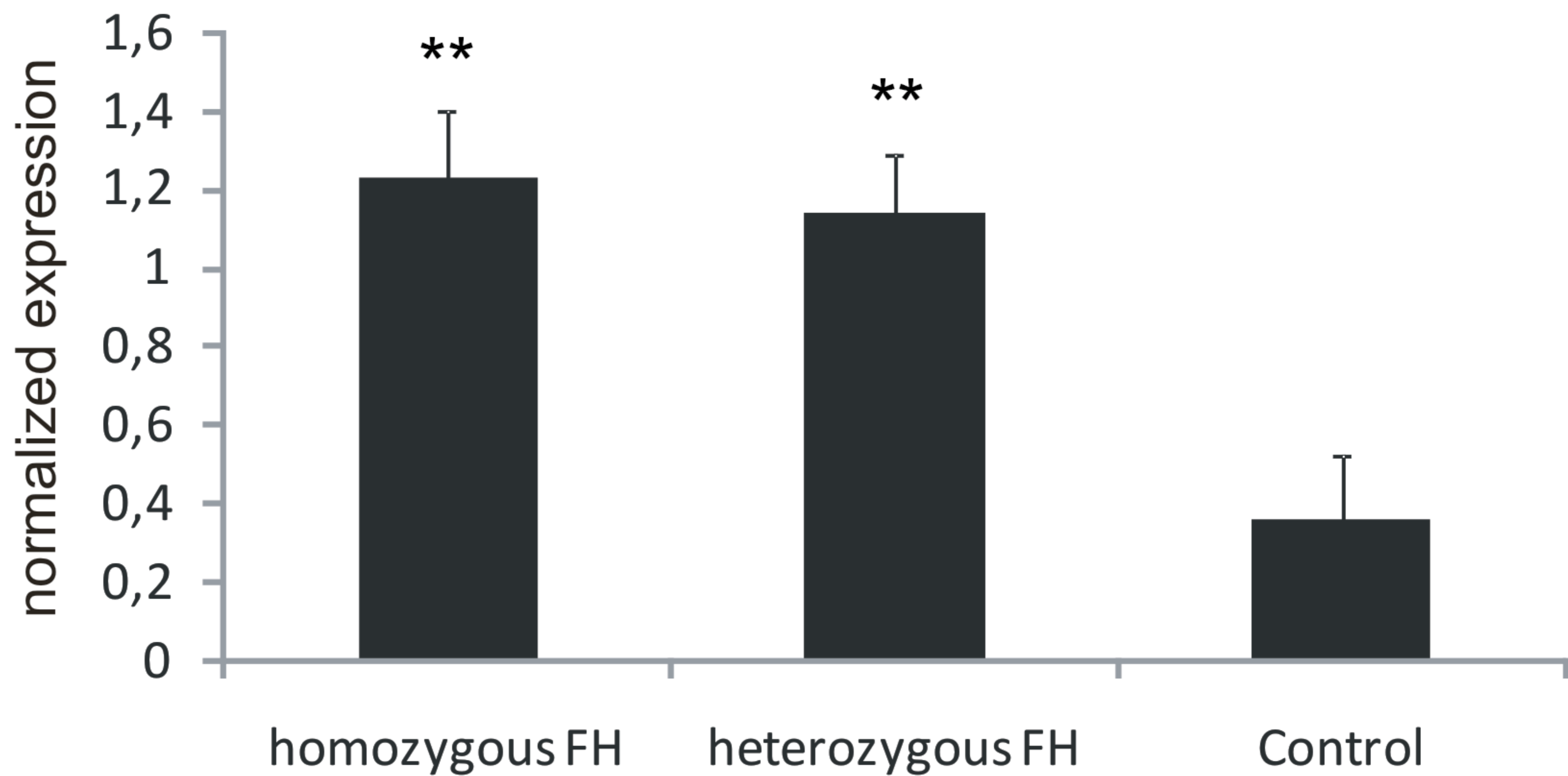

Supplement: Additional file 3 — Analysis of gene expression by qRT-PCR for CD36 and LRP1. mRNA expression of A: CD36 and B: LRP1 is elevated in monocytes of patients with homozygous or heterozygous FH compared to controls. Histogram bars indicate the mean ± SD (* p < 0.05, ** p < 0.01). [file 1755-8794-1-60-S3.pdf]

A

CD36

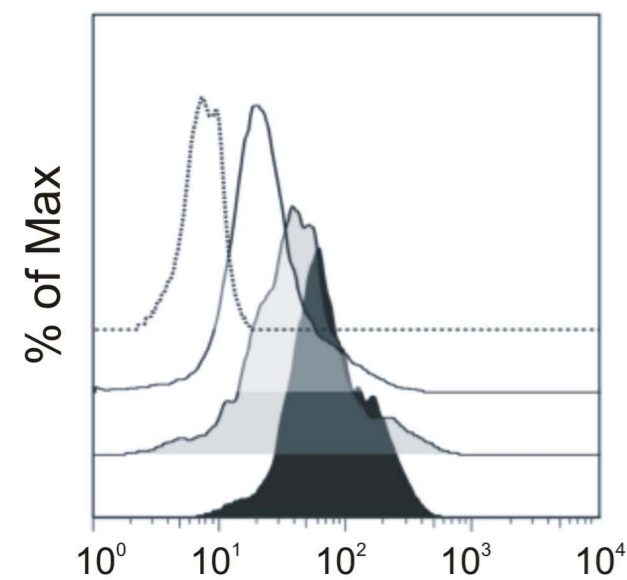

Lrp1

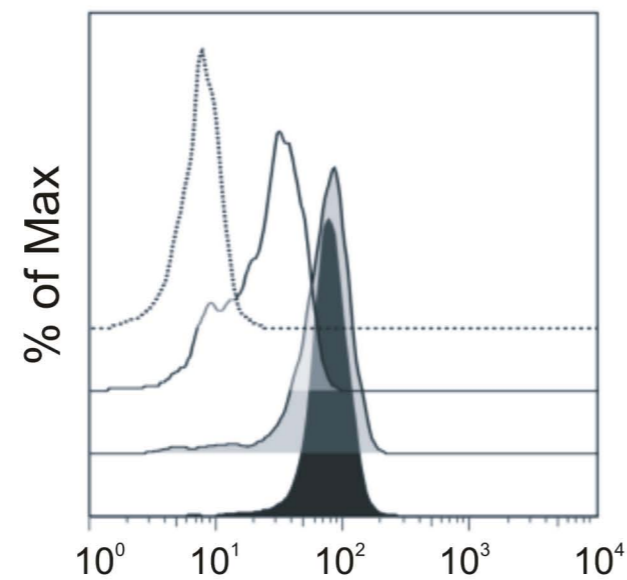

B

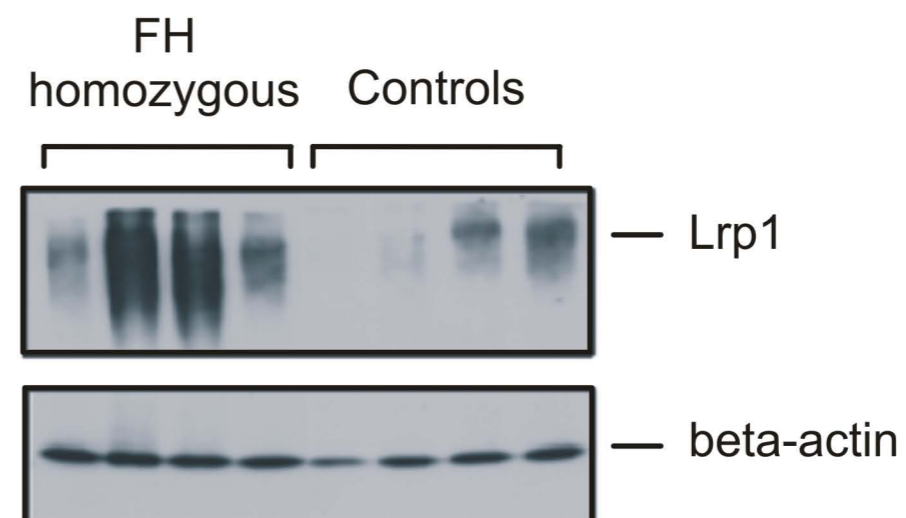

C

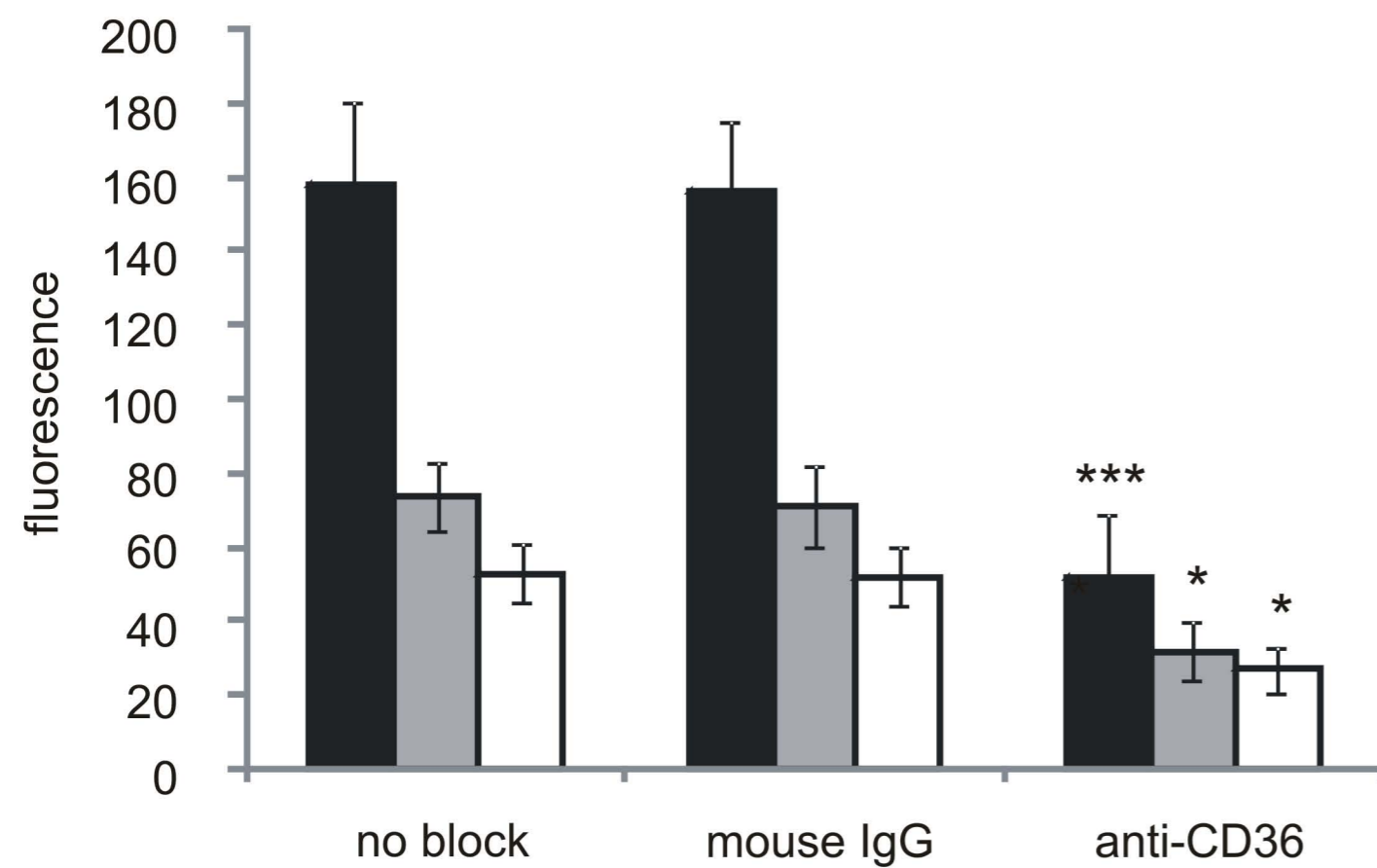

D

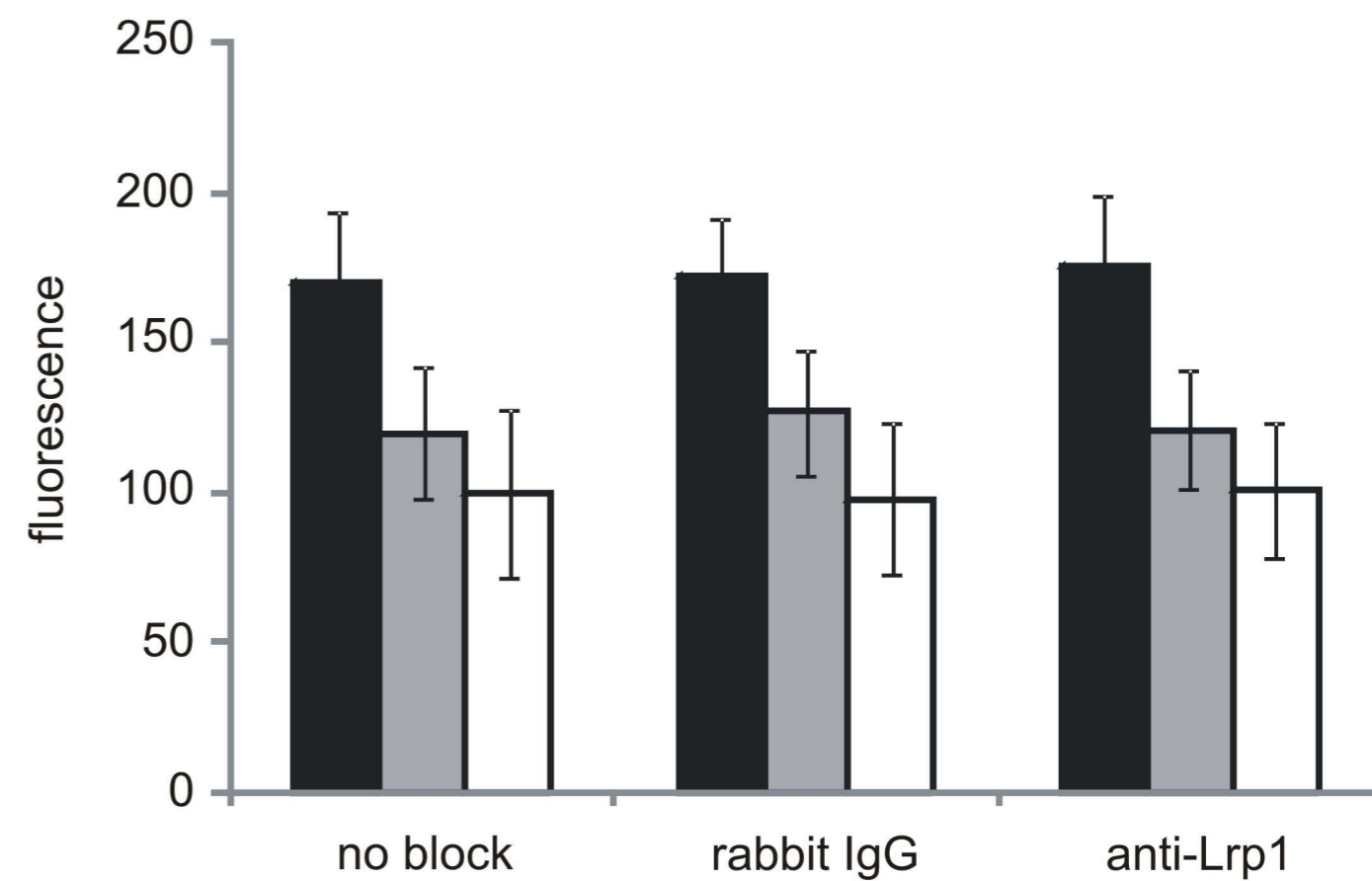

Supplement: Additional file 4 — Increased CD36 and CD91/Lrp1 levels on the surface of monocytes from FH patients and their role in LDL uptake. Figure 4A–D in this file shows increased expression of CD36 and CD91/Lrp1 on monocytes of FH patients. CD36 mediates uptake of oxLDL in monocytes of FH patients and healthy individuals. CD91/Lrp1 is not involved in nLDL uptake. [file 1755-8794-1-60-S4.pdf]

A

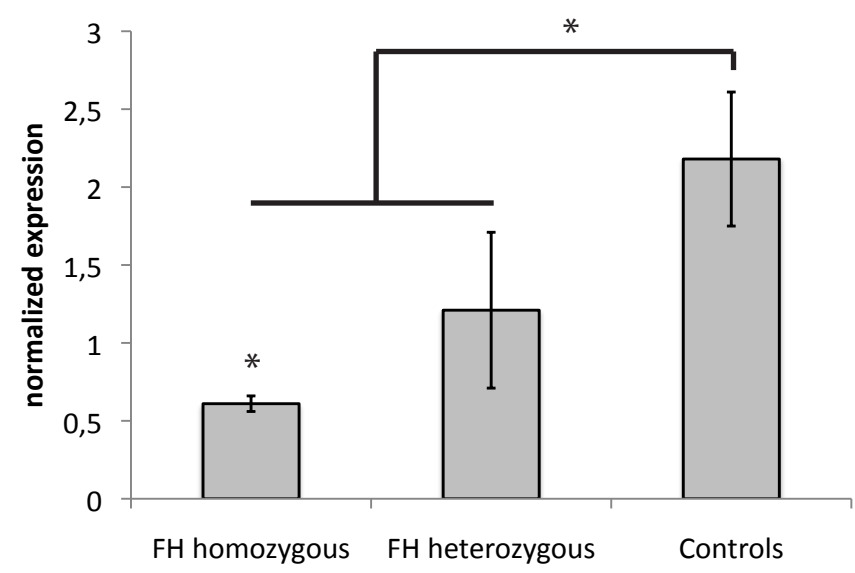

B

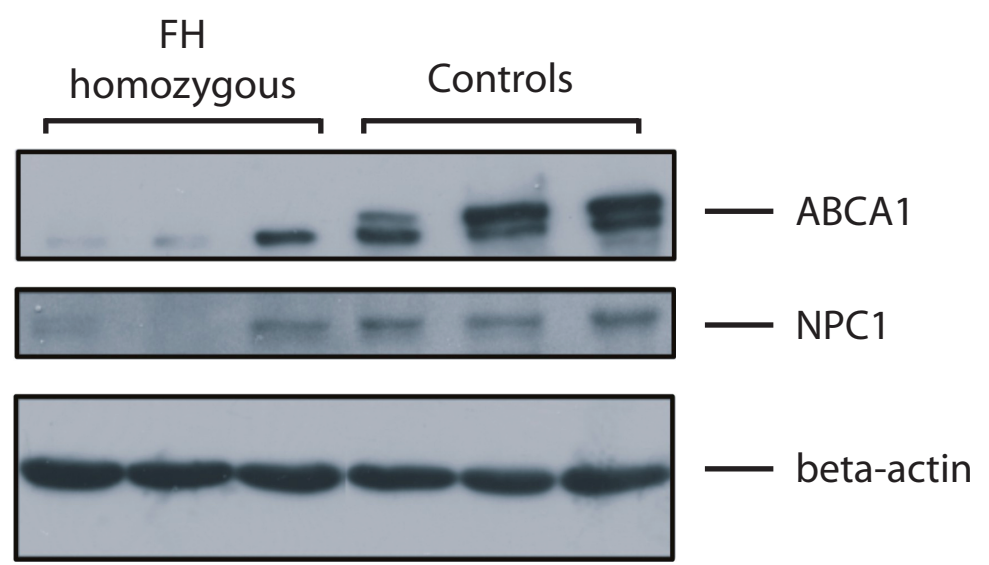

C

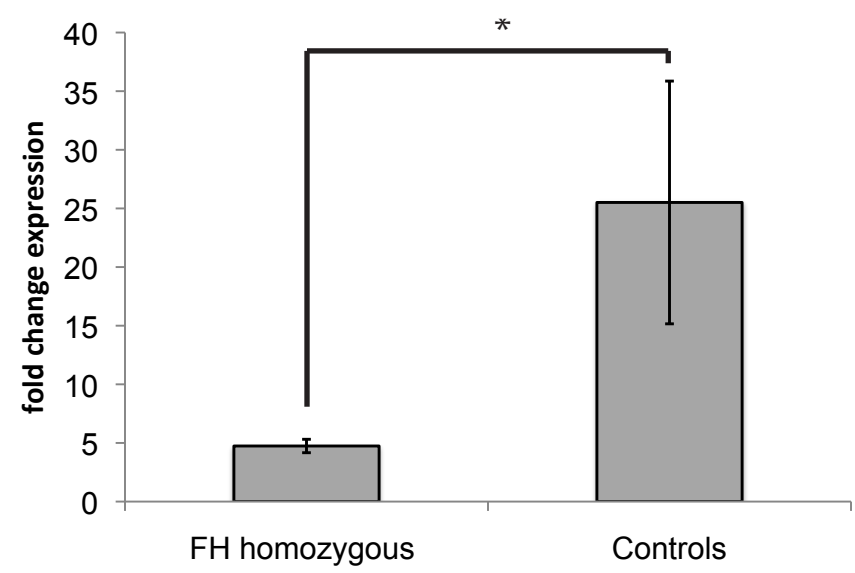

Supplement: Additional file 5 — Expression of NPC1 and ABCA1 in monocytes of FH patients and of healthy control individuals. The data provided show that NPC1 as well as ABCA1 expression is deceased in FH monocytes. ABCA1 expression is further decreased during monocyte to macrophage differentiation of FH monocytes compared to monocytes of healthy individuals. [file 1755-8794-1-60-S5.pdf]

A

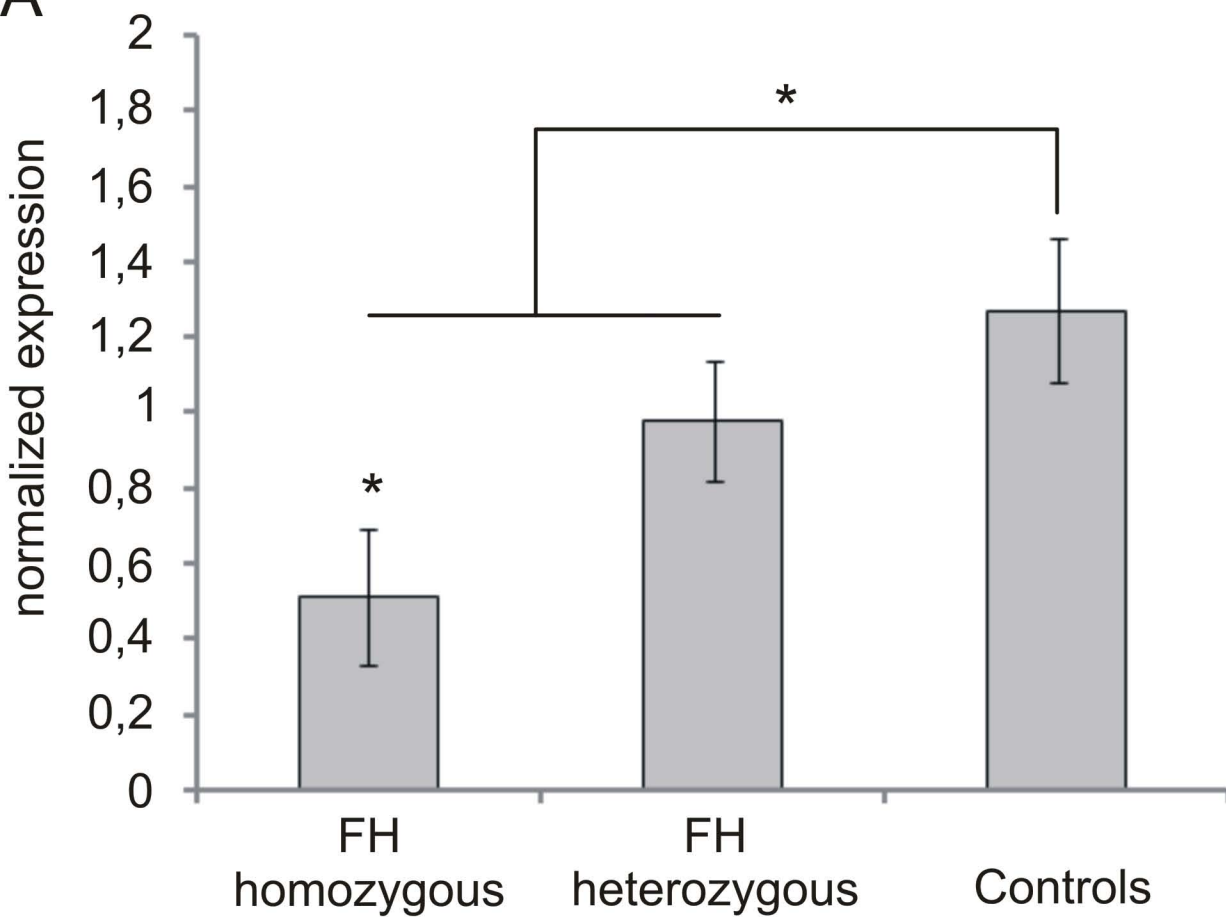

B

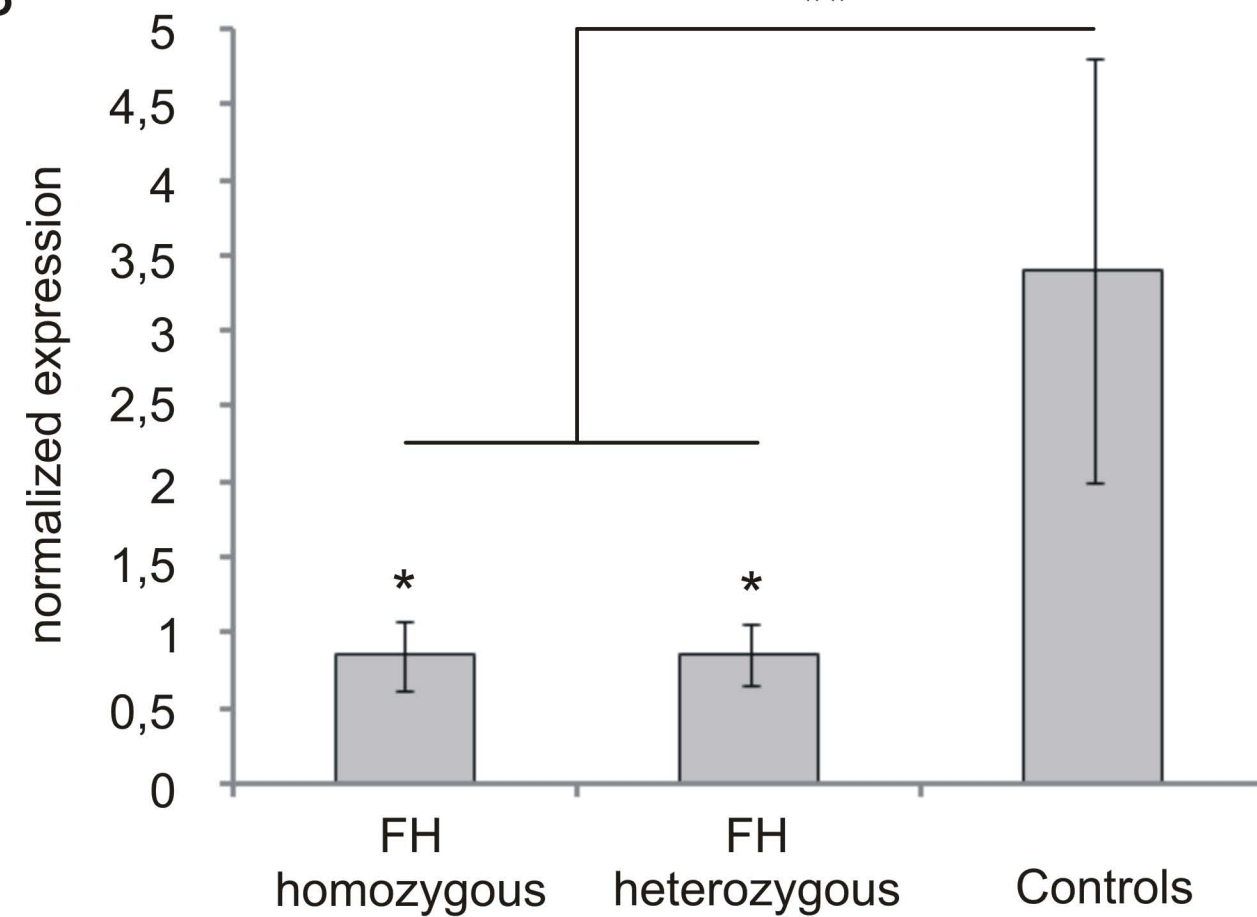

C

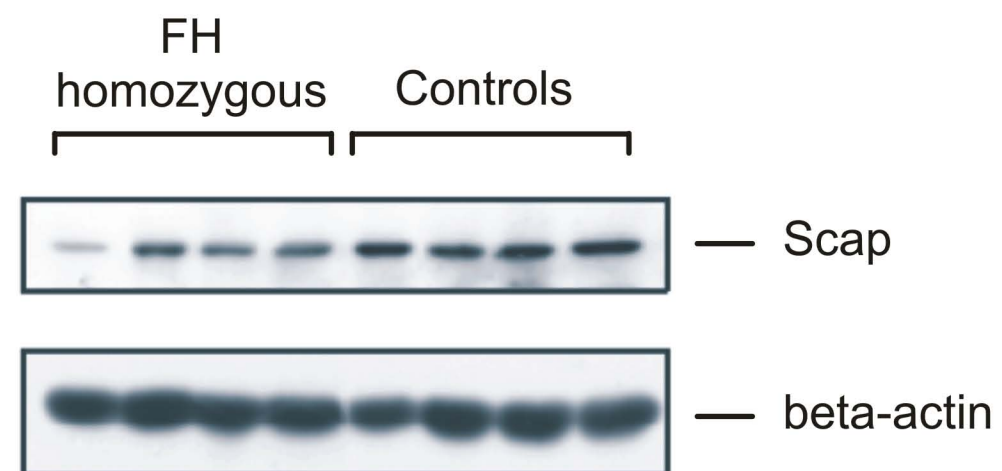

D

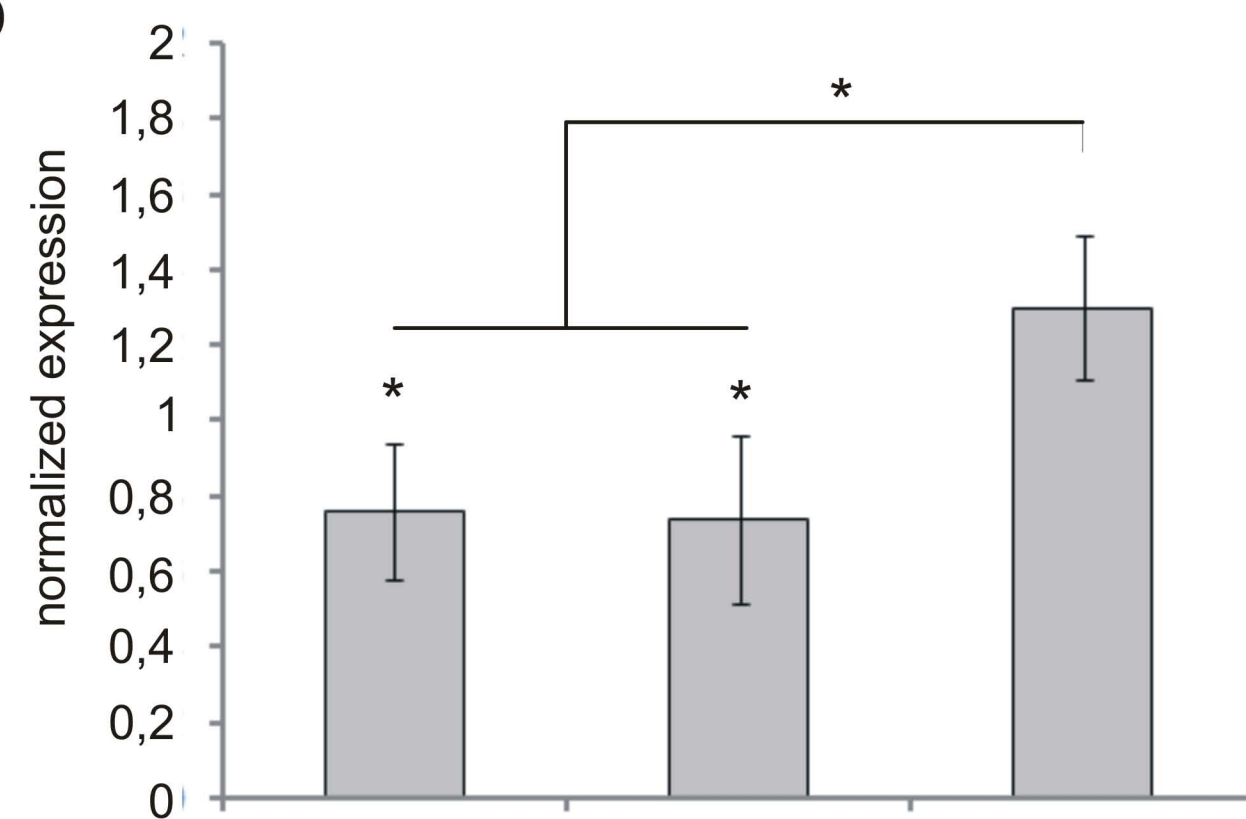

Supplement: Additional file 6 — Analyses of gene and protein expression by qRT-PCR and Western Blot reveal down-regulation of members of the SREBP-pathway. Genes and proteins of the SREBP pathway are down-regulated in monocytes of FH patients. [file 1755-8794-1-60-S6.pdf]

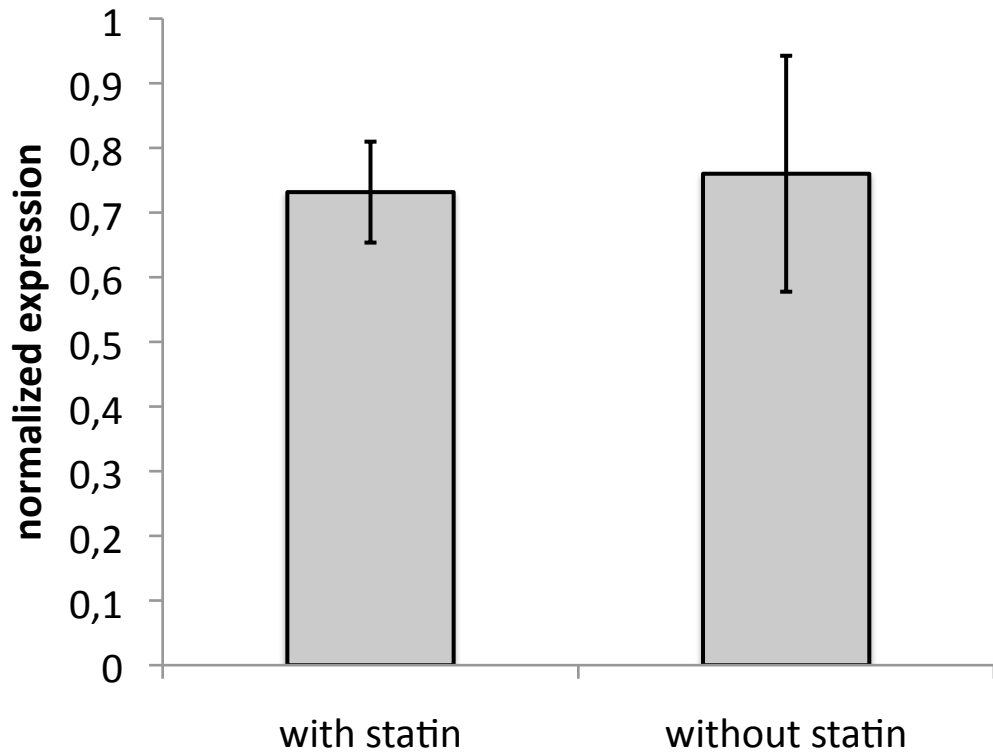

Supplement: Additional file 7 — qRT-PCR analysis of SREBP gene expression of FH patients receiving statin medication and FH patients without statin therapy. Statin therapy has no effect of SREBP gene expression in monocytes. Histogram bars indicate the mean ± SD [file 1755-8794-1-60-S7.pdf]
