# Supplementary material for: Monocytes of patients with familial hypercholesterolemia show alterations in cholesterol metabolism
Source: BMC Med Genomics. 2008 Nov 28;1:60. doi: 10.1186/1755-8794-1-60 (PMC2633353; doi:10.1186/1755-8794-1-60)
Supplement: Additional file 2 — Quality control of microarry raw data. A: legend of array index. B: Whisker box plots of log2 transformed microarray raw data. The centers of the boxes represent the median of the genes, upper and lower ends of the boxes represent the 75th and 25th quartiles, respectively. Upper whisker shows the 90th, the lower whisker the 10th percentile. C: Density plot of log2 transformed raw data. D: Plot of 3'/5' GAPDH and beta-actin ratios. Numbers behind the sample names represent % of present calls and average background, respectively. The blue line indicates the scaling factor. E: Array vs. array intensity correlation plot. [file 1755-8794-1-60-S2.pdf]

A

Array Index	Array Name
1	Control 1.CEL
2	Control 10.CEL
3	Control 11.CEL
4	Control 12.CEL
5	Control 13.CEL
6	Control 14.CEL
7	Control 15.CEL
8	Control 2.CEL
9	Control 3.CEL
10	Control 4.CEL
11	Control 5.CEL
12	Control 6.CEL
13	Control 7.CEL
14	Control 8.CEL
15	Control 9.CEL
16	FH heterozygous 1.CEL
17	FH heterozygous 2.CEL
18	FH heterozygous 3.CEL
19	FH heterozygous 4.CEL
20	FH heterozygous 5.CEL
21	FH heterozygous 6.CEL
22	FH homozygous 1.CEL
23	FH homozygous 2.CEL
24	FH homozygous 3.CEL
25	FH homozygous 4.CEL

B

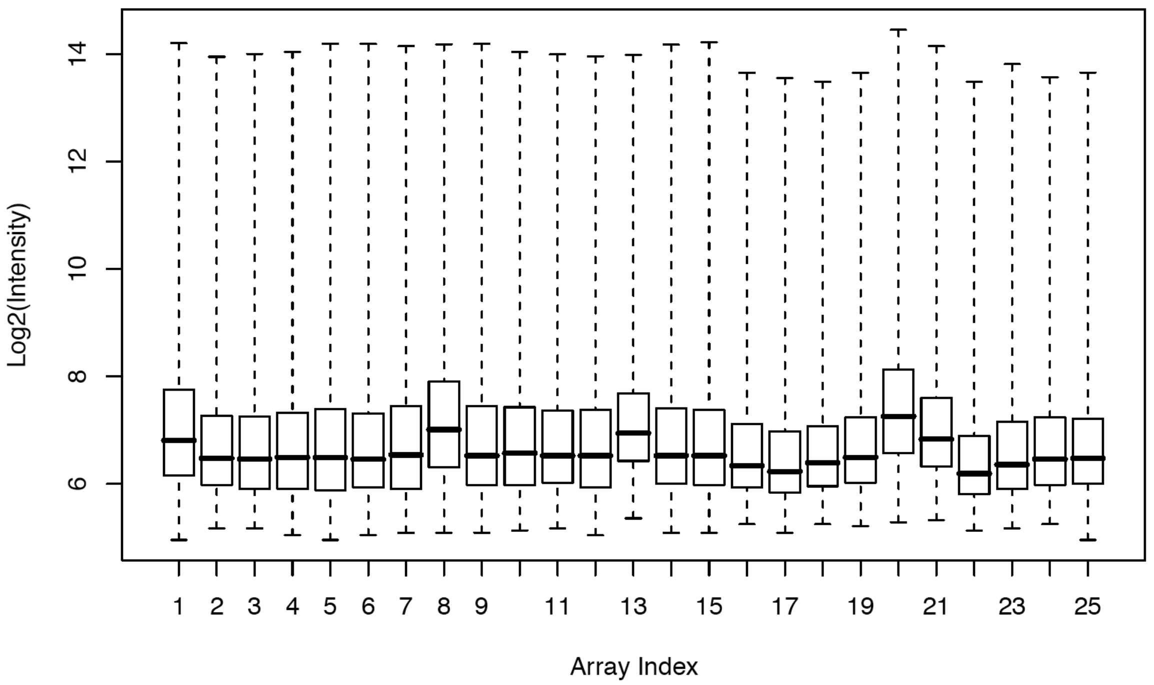

C

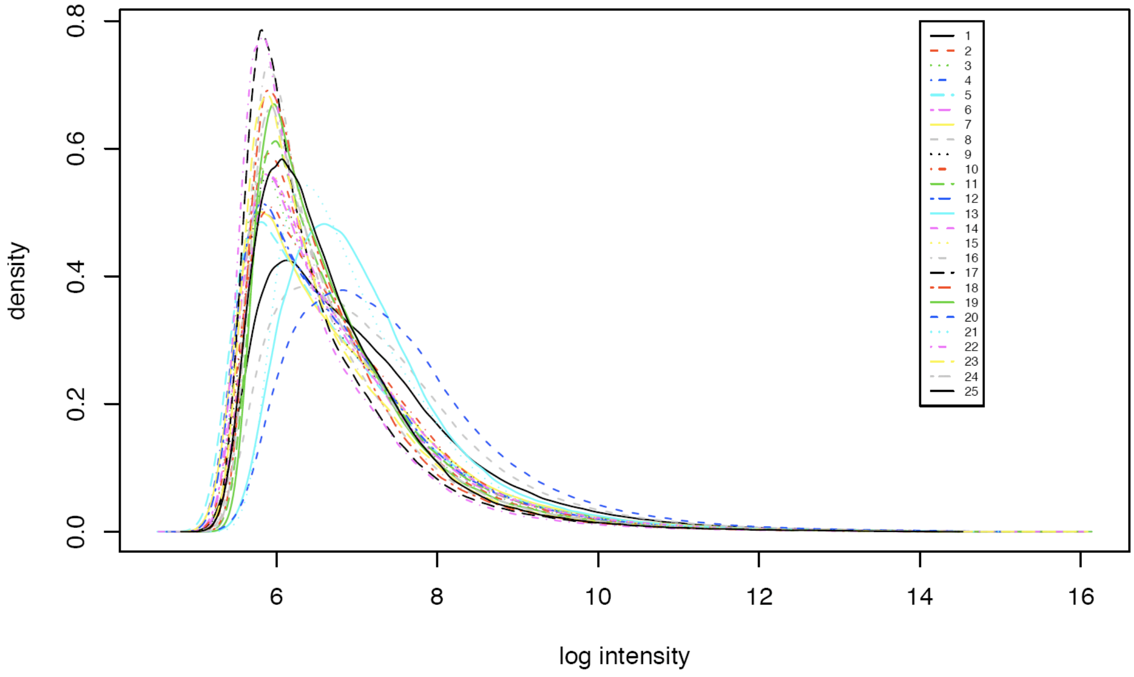
density
0.0
0.2
0.4
0.6
0.8
6
8
10
12
14
16
log intensity
1
2
3
4
5
6
7
8
9
10
11
12
13
14
15
16
17
18
19
20
21
22
23
24
25

D

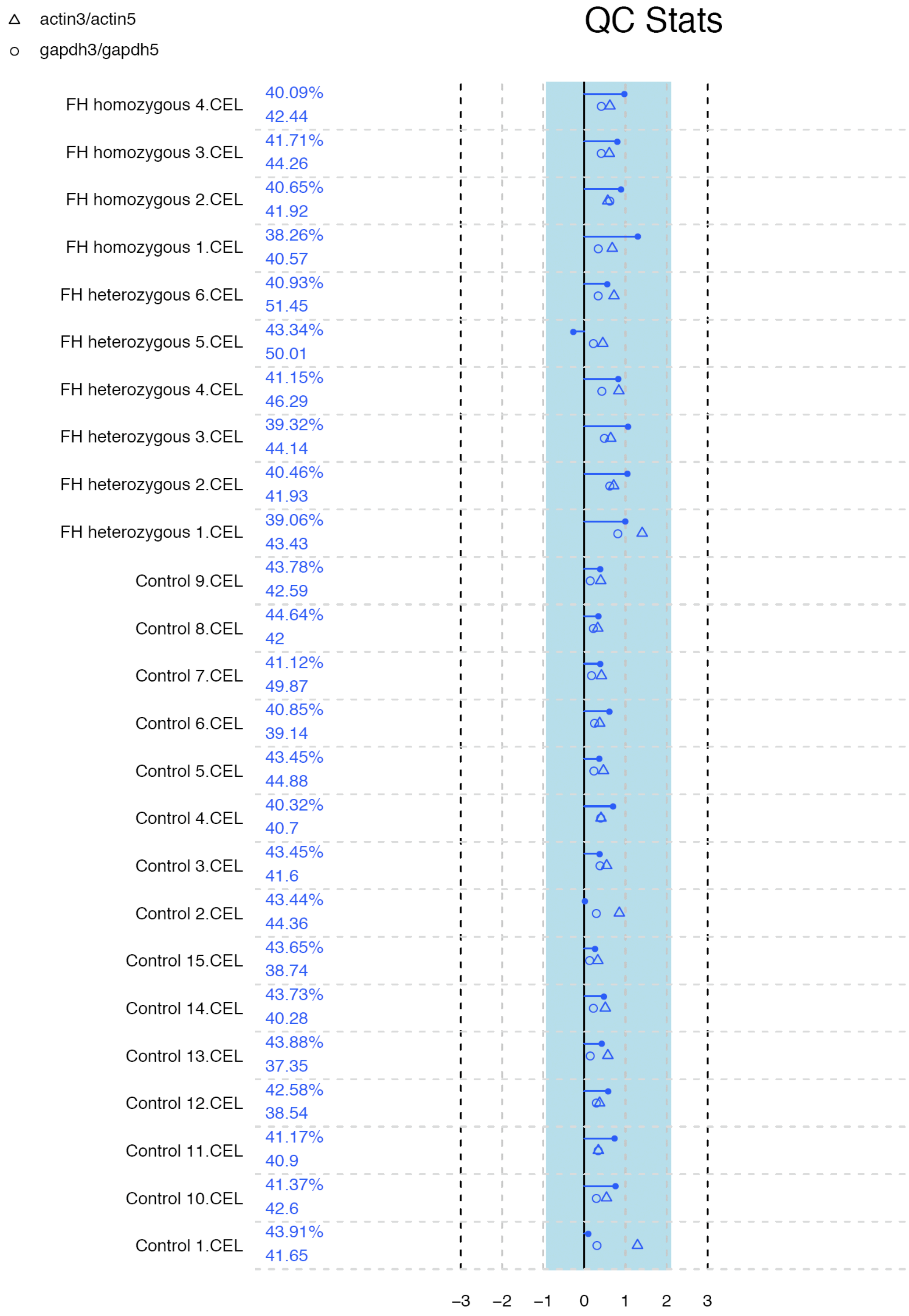
actin3/actin5
gapdh3/gapdh5
QC Stats
FH homozygous 4.CEL
40.09%
42.44
FH homozygous 3.CEL
41.71%
44.26
FH homozygous 2.CEL
40.65%
41.92
FH homozygous 1.CEL
38.26%
40.57
FH heterozygous 6.CEL
40.93%
51.45
FH heterozygous 5.CEL
43.34%
50.01
FH heterozygous 4.CEL
41.15%
46.29
FH heterozygous 3.CEL
39.32%
44.14
FH heterozygous 2.CEL
40.46%
41.93
FH heterozygous 1.CEL
39.06%
43.43
Control 9.CEL
43.78%
42.59
Control 8.CEL
44.64%
42
Control 7.CEL
41.12%
49.87
Control 6.CEL
40.85%
39.14
Control 5.CEL
43.45%
44.88
Control 4.CEL
40.32%
40.7
Control 3.CEL
43.45%
41.6
Control 2.CEL
43.44%
44.36
Control 15.CEL
43.65%
38.74
Control 14.CEL
43.73%
40.28
Control 13.CEL
43.88%
37.35
Control 12.CEL
42.58%
38.54
Control 11.CEL
41.17%
40.9
Control 10.CEL
41.37%
42.6
Control 1.CEL
43.91%
41.65
−3
−2
−1
0
1
2
3

E

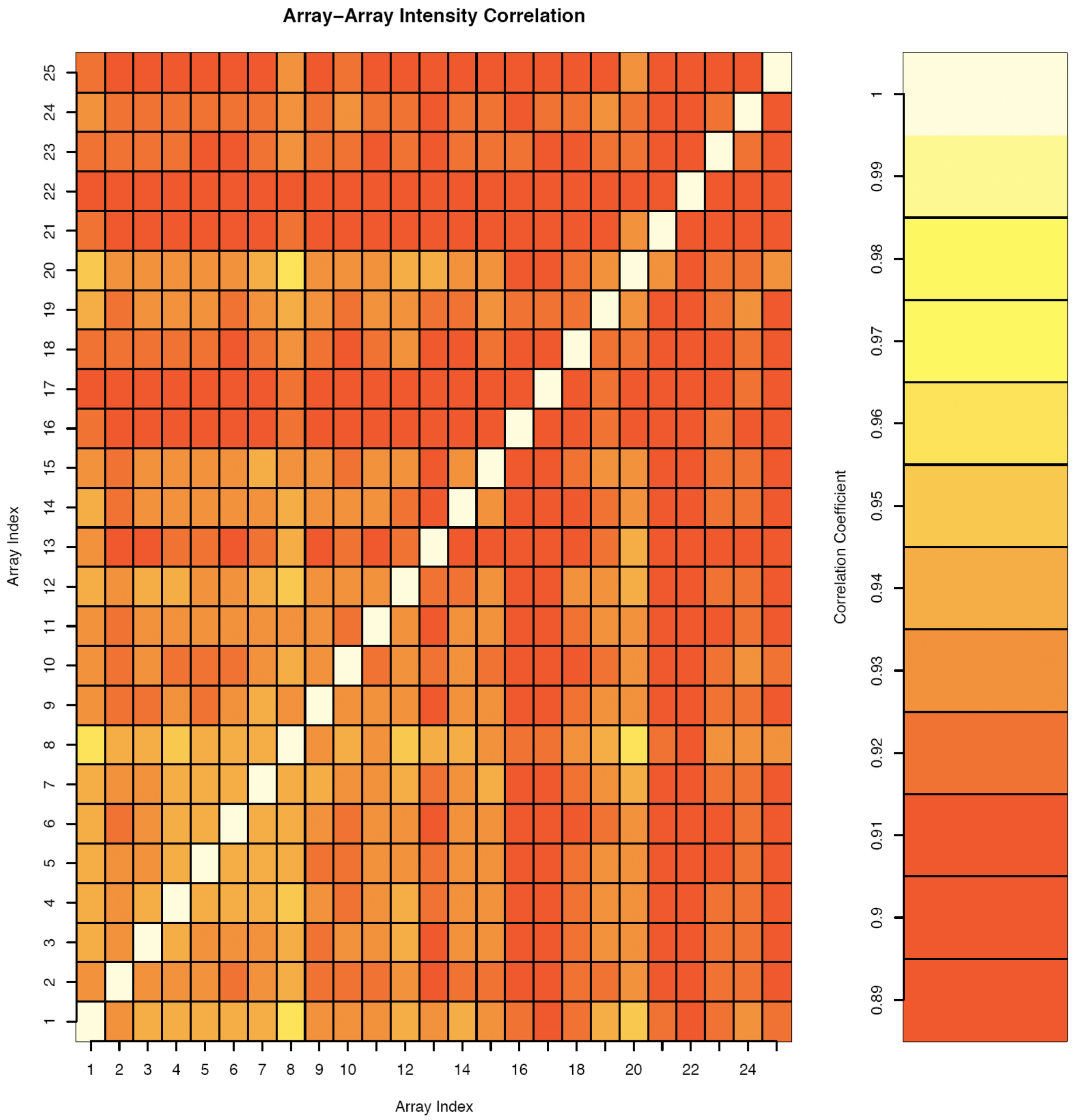

Array–Array Intensity Correlation
Array Index
Array Index
Correlation Coefficient
1
0.99
0.98
0.97
0.96
0.95
0.94
0.93
0.92
0.91
0.9
0.89
